# A FRET-Based Assay for Assessing Covalent Warhead Reactivity

**DOI:** 10.1021/acsomega.5c12902

**Published:** 2026-06-24

**Authors:** Anna P. Valaka, Carlos Benitez-Martin, Joakim Andréasson, Morten Grøtli

**Affiliations:** † Department of Chemistry and Molecular Biology, 3570 University of Gothenburg, 405 30 Gothenburg, Sweden; ‡ Department of Chemistry and Chemical Engineering, Chalmers University of Technology, 412 96 Gothenburg, Sweden

## Abstract

Covalent modalities have become increasingly important in drug discovery, yet reliable methods for evaluating the intrinsic reactivity of electrophilic warheads toward different nucleophiles remain a challenge. While Förster resonance energy transfer (FRET)-based fluorescent sensors are widely exploited in chemical biology for the detection of diverse analytes, their potential for probing covalent warhead reactivity has not been explored. Herein, we report the design and application of a FRET-based fluorescent platform for assessing covalent warhead reactivity toward biological nucleophiles. The system incorporates a sulfone-based nucleophilic aromatic substitution (S_N_Ar) warhead bridging a coumarin fluorescent donor and a dinitrophenyl dark quencher, forming a FRET dyad. The dyad operates as a reaction-based fluorescent sensor, in which S_N_Ar reaction disrupts FRET and allows for fluorescence readout, quantifying covalent warhead reactivity and demonstrating strong selectivity for thiols. The assay is performed in a 96-well plate format, which enables high-throughput screening of nucleophiles and reaction conditions. This versatile approach provides a robust tool for the evaluation of covalent warhead reactivity, facilitating mechanistic studies in chemical biology and drug discovery.

## Graphical abstract

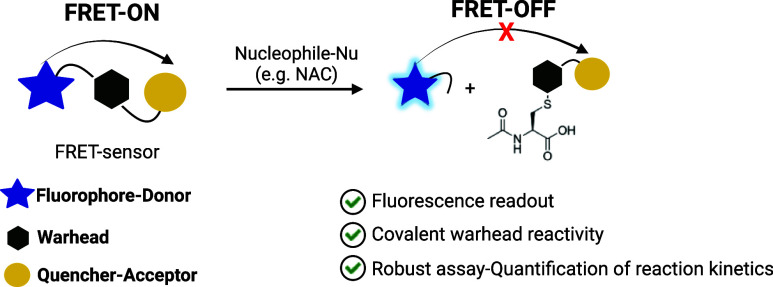

## Introduction

Protein modification through covalent modalities has emerged over the past decade as a key strategy in both medicinal chemistry and chemical biology. Targeted covalent inhibitors (TCIs), which engage poorly conserved, non-catalytic amino acid residues, have proven successful in drug discovery programs in recent years, particularly in oncology and the protein kinase research field.^1^ TCIs involve coupling a targeting ligand recognizing the protein’s active site with a reactive electrophilic group, known as a warhead. Upon non-covalent binding of the targeting ligand to the active site of the protein, the warhead reacts rapidly with a non-catalytic reactive residue.^2^

Apart from kinases, TCIs have also been developed against other protein classes, including KRAS (Kirsten rat sarcoma virus)^1^ and SARS-CoV-2.^3^ Covalency has been a major pillar in chemical biology, particularly in the field of activity-based protein profiling (ABPP). ABPP employs small-molecule probes that covalently label the active sites of enzymes, enabling functional interrogation of enzyme families such as serine hydrolases, proteases, glycosidases, cytochrome P450s, and phosphatases.^4^

Bioconjugation chemistry commonly targets amino acid residues such as cysteine, lysine, and serine. Among these, cysteine enjoys preference due to its low abundance and high reactivity as a nucleophile toward a wide range of electrophiles, compared to the rest of the amino acids.^1^

Several reactive groups have been used as warheads in the development of covalent modalities. Nucleophilic aromatic substitution (S_N_Ar) warheads represent a relatively new and sparsely explored class of electrophiles for covalent protein targeting. In S_N_Ar reactions, the nucleophile displaces a leaving group from an electron-deficient (hetero)­aryl ring. S_N_Ar warheads offer several advantages, including tunable reactivity, high selectivity for cysteine, structural rigidity, favorable metabolic stability, and straightforward synthetic accessibility.^5^ Unlike Michael acceptors, S_N_Ar warheads do not react with oxidized cysteine species such as sulfenic acids (–SOH) or *S*-nitrosothiols (–SNO),^6^ providing improved selectivity in biological environments. Altogether, these features make them an emerging and versatile class of electrophiles for covalent protein targeting. Although most S_N_Ar electrophiles employ halides as leaving groups (LGs),^7–13^ heteroaryl sulfoxides and sulfones have emerged as alternative leaving groups.^14^ Systematic investigations of the structure–reactivity relationship of these electrophiles have increased in recent years^15,16^ suggesting high selectivity toward cysteine versus other amino acids. However, strongly electron-deficient (hetero)­arenes have been reported as suitable scaffolds for targeting lysine residues in a chemoselective manner.^13,17^

Assessing the warhead’s intrinsic reactivity toward target amino acids is typically performed in solution, where the respective electrophile is tested toward protected amino acids (*N*-acetyl cysteine-NAC, *N*_a_-acetyl lysine, etc.) or small peptides such as glutathione (GSH). These assays are usually performed at physiological pH, although several lysine reactivity assays have been reported at higher pH values to mimic p*K*_a_-perturbation that can occur in the protein microenvironment.^18^ Most reactivity measurements are often carried out using HPLC^19,20^ or NMR spectroscopy,^16,21^ whereas high-throughput approaches are less common.^22–25^ By employing colorimetric or fluorogenic reagents for thiol quantification, such as the Ellman’s reagent,^22^ these methods have seen extensive use in fragment-based screening. Nevertheless, they measure electrophile reactivity indirectly through thiol consumption, thereby limiting the scope to cysteine nucleophiles.^23,24^

Fluorescence sensing and imaging offer a unique and valuable approach for detecting specific analytes in biological environments. One of the most exploited mechanisms for the generation of fluorescence sensors is Förster resonance energy transfer (FRET).^26^ FRET is a nonradiative process in which a FRET donor transfers the excitation energy to a FRET acceptor through long-range dipole–dipole interactions.^27^ The overall effect of this process is that the FRET donor is deactivated non-radiatively, rendering it non-fluorescent, and the FRET acceptor is promoted to the excited state. The efficiency of the energy transfer, or FRET-efficiency, is primarily governed by the spectral overlap (*J*_(λ)_) between the donor and the acceptor, as well as the interchromophore distance (*R*).

Although fluorescent probes for cysteine and glutathione have been extensively developed, including turn-on fluorescent,^28,29^ ratiometric^30,31^ and FRET-based probes,^32–35^ these probes are typically designed for analyte detection rather than for systematic evaluation of electrophile reactivity. To our knowledge, the use of the FRET-mechanism as a general assay platform to quantify covalent warhead reactivity toward different nucleophiles in a high-throughput format has not been explored. To address this gap, we designed a turn-on fluorescent sensor incorporating a sulfone-based S_N_Ar warhead within a FRET framework to assess electrophile reactivity toward various amino acids and GSH (Figure 1). In this design, the warhead functions as a molecular bridge between a fluorophore (FRET-donor) and a quencher (FRET-acceptor). Upon reaction with a nucleophile, the quencher is cleaved and FRET is no longer operative, thus allowing detection of the donor’s emission. The assay was optimized for a 96-well plate format, enabling simultaneous high-throughput screening of nucleophiles and buffer conditions. Rather than serving as a biological sensor, this approach functions as a reaction-based assay that enables comparative evaluation of warhead reactivity across multiple nucleophiles, an analysis not readily achievable with conventional fluorescent probes optimized for specific analytes.

**Figure 1:**
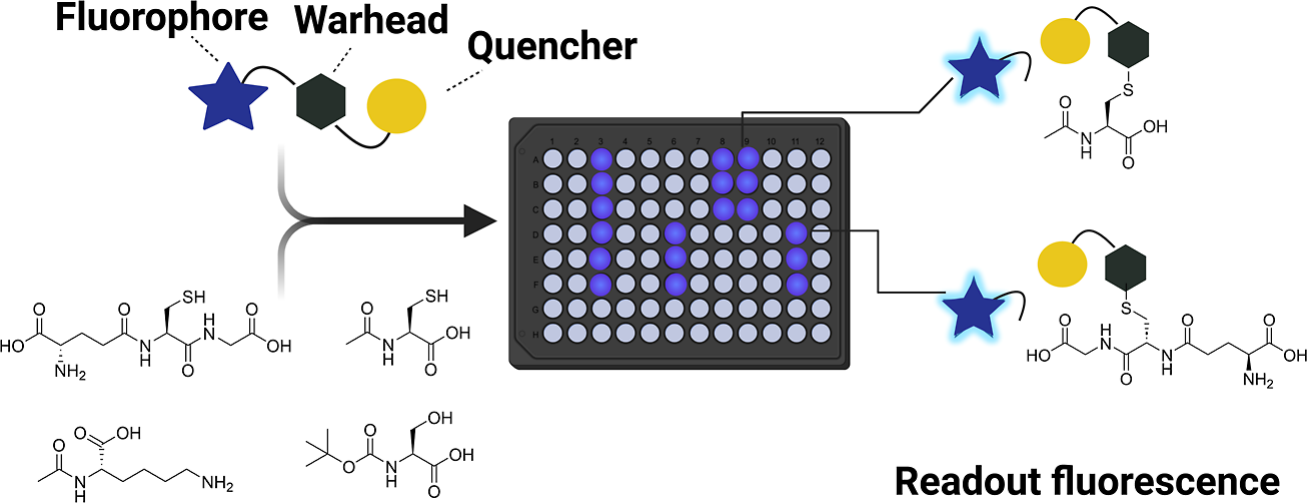
Schematic representation of a FRET-based assay. The figure was created with https://Biorender.com

In this study, we describe the design, synthesis, and validation of a FRET-based platform and demonstrate its utility for quantitative profiling of covalent warhead reactivity under physiologically relevant conditions.

## Experimental Section

All solvents and reagents were obtained from commercial suppliers, stored as indicated by the supplier, and used without further purification unless otherwise stated. Dry THF and DCM were obtained from a solvent purification system (PS-MD-5/7 Inert technology). Reactions were monitored by TLC, LC–MS, and/or HPLC. ^1^H NMR and ^13^C NMR spectra were recorded on a 600 MHz Bruker Avance Neo, or an 800 MHz Bruker Avance III HD spectrometer. All measurements were performed at 25 °C unless stated otherwise. All chemical shifts (δ) (^1^H, ^13^C) are reported in parts per million (ppm) relative to the residual solvent peak (CDCl_3_: 7.26 ppm, 77.16 ppm; (CD_3_)_2_SO: 2.50 ppm, 39.52 ppm; (CD_3_)_2_CO: 2.05 ppm, 29.84 ppm; D_2_O: 4.79 ppm). The following abbreviations are used to denote the multiplicities: s = singlet, br s = broad singlet, d = doublet, dd = doublet of doublets, t = triplet, td = triplet of doublets, tt = triplet of triplets, q = quartet, qd = quartet of doublets, m = multiplet. Coupling constants (*J*) are reported in Hz. TLC was conducted on silica-gel-coated aluminium sheets for normal-phase (Merck TLC Silica gel 60 F_254_) respectively reverse-phase (Merck TLC Silica gel 60 RP-18 F_254_s) and were visualized by UV light (λ = 254 nm or 366 nm). LC–MS was performed on a Waters Acquity system (Acquity Arc HPLC system; 2489 UV/Vis Detector; XBridge BEH C18 column, 130 Å, 2.5 μm, 2.1 × 50 mm; XBridge BEH C18 Guard column, V–Gd Cart 2.5 μm, 2.1 × 5 mm; Acquity QDa Mass Detector; MeCN/water (0.01% formic acid), 40 °C). Analytical HPLC was performed on a Waters system (2690 Separation Module; 996 Photodiode Array Detector; Chromolith SpeedROD RP-18 endcapped 50–4.6 HPLC column; MeCN/water (0.1% TFA)). Preparative HPLC was performed on a Waters system (1525 Binary HPLC Pump; 2998 Photodiode Array Detector; Atlantis Prep T3 OBD column; MeCN/water (0.1% TFA)), by injecting the crude dissolved in MeOH. Column chromatography was performed on a Selekt or Isolera One flash chromatography system (Biotage) for normal-phase or reverse-phase, respectively. The silica gel was Sfär Silica D Duo 60 μm or Sfär KP-Amino D Duo 50 μm cartridges for normal-phase, and Sfär C18 D Duo 100 Å 30 μm cartridges for reverse-phase (Biotage). For all column chromatography, dry loading was performed using the same type of silica as the stationary phase. The reactions that employed microwave irradiation were performed in capped vials using an initiator + microwave synthesizer (Biotage) with fixed hold time. HRMS data were recorded with a QExactive HF Orbitrap mass spectrometer interfaced with Dionex Ultimate 3000 liquid chromatography system (Thermo Fisher Scientific). The instrument operated in full MS mode only, where the ion mass spectra were acquired at a resolution of 120,000, maximum injection time 200 ms for 3 × 10^6^ ions. The Orbitrap was calibrated with Pierce LTQ ESI positive ion calibration solution prior to the analysis, resulting in a mass accuracy better than 5 ppm. Electrospray ionization was performed at 4 kV and 320 °C using a metal emitter in the ion source. The sample (1 or 10 μL) was injected onto a reversed-phase XBridge BEH C18 column (3.5 μm, 2.1 × 50 mm, Waters). The analysis was performed using a linear gradient over 2.5 min from 10 to 100% solvent B, followed by isocratic eluted with 100% solvent B for 17.5 min with a flow of 0.300 mL/min (solvent A: water with 0.1% formic acid; solvent B: 80% acetonitrile in water with 0.1% formic acid). Data analysis was performed using the Xcalibur software (Thermo Fisher Scientific).

### Chemical Synthesis

#### Synthesis of 7-Methoxy-2-oxo-2*H*-chromene-3-carboxylic acid (**2**)

Compound **2** was synthesized according to a literature procedure by Yao et al.^36^

#### Synthesis of *tert*-Butyl (2-((2,4-dinitrophenyl)­amino)­ethyl)­carbamate (**4a**)

A round-bottom flask (100 mL) equipped with a stir bar was charged with 2,4-dinitro-1-fluorobenzene (638 mg, 3.42 mmol, 1.0 equiv) in THF (6 mL). Then a solution of *N*-Boc-ethylenediamine (597 μL, 3.77 mmol, 1.10 equiv) and DIPEA (956 μL, 6.85 mmol, 2.0 equiv) in THF (1 mL) was added in one portion. The reaction mixture was stirred at room temperature until TLC showed full consumption of the starting material. After 2 h, solvent was evaporated, and the crude was diluted with DCM (25 mL) and washed with saturated NH_4_Cl (2 × 10 mL) and brine (10 mL). The organic layer was dried over anhydrous Na_2_SO_4_ and concentrated under reduced pressure. The residue was loaded onto silica and purified by flash column chromatography (Biotage, SNAP 25 g, Pent/EtOAc, 0–60%), to afford compound **4a** as a yellow solid, (746 mg, 67% yield). ^1^H NMR (600 MHz, CDCl_3_): δ 9.15 (d, *J* = 2.7 Hz, 1H), 8.75 (s, 1H), 8.29 (ddd, *J* = 9.5, 2.7, 0.7 Hz, 1H), 7.04 (d, *J* = 9.5 Hz, 1H), 4.83 (s, 1H), 3.58 (q, *J* = 5.9 Hz, 2H), 3.48 (q, *J* = 6.1 Hz, 2H), 1.46 (s, 9H). ^13^C NMR (151 MHz, CDCl_3_): δ 156.6, 148.9, 136.7, 131.1, 130.7, 124.6, 114.3, 80.7, 44.0, 39.7, 28.7. **HRMS (ESI)** *m*/*z*: [M + Na]^+^ calcd. for C_13_H_18_N_4_O_6_, 349.1118; found, 349.1119.

#### Synthesis of 2-((2,4-Dinitrophenyl)­amino)­ethan-1-aminium chloride (**Q-amine**)

A round-bottom flask (25 mL) equipped with a stir bar was charged with **4a** (297 mg, 0.91 mmol, 1 equiv) was dissolved in MeOH (3 mL), cooled on ice, and purged with N_2_. HCl 4 M in 1,4-dioxane (2.2 mL, 9.1 mmol, 10 equiv) was added dropwise and the yellow solution was stirred at room temperature. After 24 h, LC–MS indicated full consumption of the starting material, the solution was concentrated in vacuo to afford **Q-amine** as a yellow solid, which was used in the next step without further purification. ^1^H NMR (600 MHz, DMSO): δ 8.90–8.83 (m, 2H), 8.29 (ddd, *J* = 9.6, 2.7, 0.7 Hz, 1H), 7.95 (s, 3H), 7.34 (d, *J* = 9.6 Hz, 1H), 3.80 (q, *J* = 5.7 Hz, 2H), 3.04 (t, *J* = 6.2 Hz, 2H). ^13^C NMR (151 MHz, DMSO): δ 148.0, 135.2, 130.7, 129.9, 123.5, 115.1, 37.2. **HRMS (ESI)** *m*/*z*: [M + H]^+^ calcd. for C_8_H_11_N_4_O_4_, 227.0774; found, 227.0772.

#### Synthesis of 2-Cyano-3-(1*H*-indol-3-yl)­prop-2-enethioamide (**5a**)

Compound **5a** was synthesized according to a literature procedure by Dyachenko, V. D. et al.^37^ in one step from indole-3-carboxaldehyde and 2-cyanothioacetamide (Scheme S1).

#### Synthesis of Ethyl 5-cyano-6-mercapto-2-methylnicotinate (**5**)

Compound **5** was synthesized according to a literature procedure by Dyachenko, V. D. et al.^38^ in two steps from **5a** (Scheme S1).

#### Synthesis of Ethyl (*R*)-6-((1-(*tert*-butoxycarbonyl)­piperidin-3-yl)­thio)-5-isocyano-2-methylnicotinate (**6**)

A microwave vial (5 mL) equipped with a stir bar was charged with **5** (383 mg, 1.72 mL, 1 equiv), (*S*)-1-Boc-3-hydroxypiperidine (520 mg, 2.58 mmol, 1.5 equiv) and cyanomethylene tributylphosphorane (CMBP) (677 μL, 2.58 mmol, 1.5 equiv) in toluene (3 mL). The vial was sealed, the mixture degassed with bubbling N_2_ for 5 min, and the reaction was stirred at 100 °C for 16 h. The reaction mixture was then diluted with EtOAc (20 mL) and washed with water (1 × 10 mL), saturated Na_2_CO_3_ (2 × 10 mL) and brine (1 × 10 mL). The organic phase was separated, dried over Na_2_SO_4_, filtered, and concentrated under reduced pressure. The crude product was purified by flash column chromatography (HP 10 g, Pent/EtOAc, 0–20%), to afford compound **6** as a yellow oil. ^1^H NMR (600 MHz, CDCl_3_): δ 8.30 (s, 1H), 4.36 (q, *J* = 7.2 Hz, 2H), 4.15–4.10 (m, 1H), 4.08–3.89 (m, 1H), 3.67–3.59 (m, 1H), 3.44–3.27 (m, 1H), 3.24 (d, *J* = 11.6 Hz, 1H), 2.86 (s, 3H), 2.16–2.07 (m, 1H), 1.85–1.70 (m, 2H), 1.64 (dtd, *J* = 9.2, 6.8, 2.7 Hz, 1H), 1.39 (dt, *J* = 14.3, 7.7 Hz, 13H). ^13^C NMR (151 MHz, CDCl_3_): δ 171.4, 165.0, 164.8, 164.1, 154.8, 143.1, 120.9, 115.2, 104.8, 61.9, 60.6, 49.2, 44.8, 41.7, 30.3, 28.6, 25.9, 21.3, 13.9. **HRMS (ESI)** *m*/*z*: [M + H]^+^ calcd. for C_20_H_27_N_3_O_4_S, 406.1794; found, 406.1788.

#### Synthesis of *tert*-Butyl (*R*)-3-((5-((2-((2,4-dinitrophenyl)­amino)­ethyl)­carbamoyl)-3-isocyano-6-methylpyridin-2-yl)­thio)­piperidine-1-carboxylate (**7**)

A round-bottom flask (10 mL) equipped with a stir bar was charged with **6** (156 mg, 0.38 mmol, 1 equiv) in ethanol (2 mL). The flask was cooled in an ice bath for 10 min, LiOH (64.5 mg, 2.69 mmol, 7 equiv) was added in one portion and the reaction mixture was allowed to warm to room temperature and was further stirred until LC–MS verified full consumption of the starting material. The mixture was then acidified with HCl 1 M, diluted with H_2_O, and the solvent was evaporated in vacuo to afford the intermediate carboxylic acid as a pale-yellow solid (140 mg, 96% yield). To a solution of the carboxylic acid (120 mg, 0.31 mmol, 1 equiv) in DMF (2 mL), NHS (47.5 mg, 0.41 mmol, 1.3 equiv) and EDCI (64 mg, 0.41 mmol, 1.3 equiv) were added at 0 C. The mixture was then stirred at ambient temperature until LC–MS confirmed the formation of the NHS-ester. Then, a solution of Q-amine 4 (72 mg, 0.31 mmol, 1 equiv) and DIPEA (60 μL, 0.34 mmol, 1.1 equiv) were added to the mixture in one portion and the reaction was stirred for 15 min until completion, as indicated by LC–MS. The crude was then diluted with DCM (15 mL) and washed with saturated NH_4_Cl (2 × 5 mL) and brine (1 × 5 mL). The organic layer was dried over anhydrous Na_2_SO_4_ and concentrated under reduced pressure to afford the crude. The residue was then purified by flash column chromatography (HP 10 g, 10% MeOH in EtOAc), to afford compound **8** as a yellow oil (35 mg, 58% yield over two steps). ^1^H NMR (600 MHz, CDCl_3_): δ 9.08 (d, *J* = 2.7 Hz, 1H), 8.81 (t, *J* = 5.5 Hz, 1H), 8.31 (dd, *J* = 9.5, 2.7 Hz, 1H), 7.83 (s, 1H), 7.10 (d, *J* = 9.5 Hz, 1H), 6.84 (s, 1H), 4.09 (dq, *J* = 7.8, 4.1 Hz, 1H), 4.00–3.90 (m, 1H), 3.80 (q, *J* = 6.1 Hz, 2H), 3.73 (q, *J* = 5.9 Hz, 2H), 3.64–3.56 (m, 1H), 3.25 (ddd, *J* = 12.8, 8.6, 3.3 Hz, 1H), 2.71 (s, 3H), 2.11 (dp, *J* = 11.0, 3.8 Hz, 1H), 1.77 (ttd, *J* = 12.9, 7.8, 3.6 Hz, 2H), 1.63 (ddd, *J* = 17.1, 8.6, 3.8 Hz, 2H), 1.47–1.35 (m, 9H). ^13^C NMR (151 MHz, CDCl_3_): δ 167.7, 161.7, 154.9, 148.7, 139.7, 136.8, 131.1, 130.9, 130.6, 125.9, 124.6, 115.5, 114.4, 114.3, 104.1, 80.1, 49.1, 48.6, 44.8, 43.4, 41.8, 39.3, 39.1, 32.2, 30.2, 30.0, 30.0, 29.7, 28.7, 27.2, 24.7, 24.4. **HRMS (ESI)** *m*/*z*: [M + Na]^+^ calcd. for C_26_H_31_N_7_O_7_S, 608.1897; found, 608.1893.

#### Synthesis of (*R*)-*N*-(2-((2,4-Dinitrophenyl)­amino)­ethyl)-5-isocyano-2-methyl-6-(piperidin-3-ylthio)­nicotinamide (**8a**)

A round-bottom flask (10 mL) equipped with a stir bar was charged with **7** (35 mg, 0.05 mmol, 1 equiv) in DCM (1 mL). The flask was cooled in an ice bath, TFA (300 μL, 15.6 mmol, 65 equiv) was added in one portion and the reaction mixture was stirred at ambient temperature for 4 h until completion, as indicated by LC–MS. Solvent was evaporated in vacuo and the crude was used in the next step without further purification (29 mg, 100%).

#### Synthesis of (*R*)-*N*-(2-((2,4-Dinitrophenyl)­amino)­ethyl)-5-isocyano-6-((1-(7-methoxy-2-oxo-2*H*-chromene-3-carbonyl)­piperidin-3-yl)­thio)-2-methylnicotinamide (**8**)

In a 5 mL MW vial, **8a** (29 mg, 0.07 mmol, 1.2 equiv) was dissolved in DMF (500 μL) and basified by the addition of DIPEA (10 μL, 0.07 mmol, 1.2 equiv). The solution was stirred at room temperature for 10 min. A 10 mL round-bottom flask was charged with **2** (11 mg, 0.05 mmol, 1 equiv), HATU (23 mg, 0.07 mmol, 1.2 equiv) and DIPEA (10 μL, 0.07 mmol, 1.2 equiv) in DMF (500 μL) and the reaction was stirred at ambient temperature for 10 min, followed by addition of the amine solution from the MW vial, and the reaction was left stirring at ambient temperature for 20 min until completion as indicated by LC–MS. Solvent was then evaporated, and the crude was purified by reversed-phase column chromatography (C18 silica 6 g, 0–100% MeCN/H_2_O) to afford **8** (eluted at 70% MeCN) as yellow oil (27 mg, 78% yield). ^1^H NMR (600 MHz, DMSO): δ 8.98 (dt, *J* = 12.3, 6.1 Hz, 2H), 8.90–8.87 (m, 2H), 8.75 (t, *J* = 5.8 Hz, 1H), 8.49 (s, 1H), 8.31–8.24 (m, 3H), 8.16 (d, *J* = 12.0 Hz, 2H), 7.72 (d, *J* = 8.7 Hz, 1H), 7.46 (s, 2H), 7.36–7.30 (m, 2H), 7.06 (d, *J* = 2.4 Hz, 1H), 7.01 (dd, *J* = 8.7, 2.5 Hz, 1H), 6.88 (dd, *J* = 16.7, 5.4 Hz, 2H), 5.01 (p, *J* = 6.2 Hz, 1H), 4.46 (dd, *J* = 16.8, 9.7 Hz, 2H), 4.08 (ddd, *J* = 22.6, 11.7, 5.8 Hz, 2H), 3.98 (s, 1H), 3.89 (s, 3H), 3.79 (s, 4H), 3.73–3.64 (m, 5H), 3.56–3.47 (m, 4H), 3.43 (dq, *J* = 12.3, 5.8 Hz, 2H), 3.02 (td, *J* = 6.6, 3.9 Hz, 3H), 2.62 (s, 3H), 2.16 (d, *J* = 35.9 Hz, 6H), 1.94–1.80 (m, 3H), 1.76–1.62 (m, 7H), 1.41–1.32 (m, 3H). ^1^H NMR showed two conformers in a 70:30 ratio. ^13^C NMR (201 MHz, DMSO): δ 169.8, 166.4, 163.8, 163.5, 163.4, 161.2, 160.8, 158.0, 157.8, 155.7, 155.3, 148.5, 143.8, 142.8, 140.8, 140.3, 135.1, 130.3, 130.2, 130.1, 129.9, 127.3, 123.8, 121.2, 120.4, 115.4, 115.4, 115.3, 113.3, 113.1, 112.1, 103.3, 103.1, 100.8, 100.4, 69.9, 56.3, 56.1, 48.1, 46.9, 46.2, 46.0, 42.6, 42.5, 41.7, 26.1, 26.1, 25.1, 23.6, 21.6, 21.5, 15.9. ^13^C showed two conformers. **HRMS (ESI)** *m*/*z*: [M + H]^+^ calcd. for C_32_H_29_N_7_O_9_S, 688.1819; found, 688.1815.

#### Synthesis of (*R*)-*N*-(2-((2,4-Dinitrophenyl)­amino)­ethyl)-5-isocyano-6-((1-(7-methoxy-2-oxo-2*H*-chromene-3-carbonyl)­piperidin-3-yl)­sulfonyl)-2-methylnicotinamide (**FRET-Dyad**)

In a 10 mL round-bottom flask, thioether **8** (27 mg, 0.04 mmol, 1 equiv) was dissolved in CHCl_3_ (500 μL) and cooled to 0 °C. Then, a solution of mCPBA (44 mg, 0.2 mmol, 5 equiv) in CHCl_3_ (1 mL) was added dropwise and the reaction mixture was then stirred at ambient temperature until LC–MS verified the consumption of the starting material. The reaction mixture was then partitioned between CHCl_3_ (10 mL) and an aqueous solution of saturated Na_2_S_2_O_3_ and Na_2_CO_3_ (5 mL, 1:1 ratio). The aqueous layer was extracted with CHCl_3_ (2 × 10 mL). The combined organic layers were washed with H_2_O (2 × 5 mL), brine (1 × 5 mL), dried over anhydrous Na_2_SO_4_, and concentrated under reduced pressure. The crude was then charged on silica and purified by preparative-HPLC (C18 column, 30–90% MeCN/H_2_O with 0.1% TFA) to afford **FRET-Dyad** (eluted at 60% MeCN, 0.1% TFA) as a yellow solid (9 mg, 33% yield). ^1^H NMR (600 MHz, DMSO): δ 8.99 (dt, *J* = 19.4, 6.0 Hz, 2H), 8.88 (d, *J* = 2.8 Hz, 1H), 8.63 (s, 1H), 8.54 (s, 1H), 8.30 (ddd, *J* = 9.5, 6.0, 3.0 Hz, 1H), 8.14 (d, *J* = 29.1 Hz, 1H), 7.66 (dd, *J* = 25.3, 8.7 Hz, 1H), 7.35 (dd, *J* = 9.2, 2.9 Hz, 1H), 7.04 (q, *J* = 2.5 Hz, 1H), 6.99 (td, *J* = 9.3, 2.4 Hz, 1H), 4.76 (d, *J* = 12.7 Hz, 1H), 4.26 (s, 1H), 3.98–3.90 (m, 1H), 3.87 (d, *J* = 3.4 Hz, 3H), 3.72 (p, *J* = 6.6 Hz, 2H), 3.65–3.52 (m, 3H), 3.23–3.11 (m, 2H), 2.69 (s, 2H), 2.42 (s, 1H), 2.22 (d, *J* = 11.6 Hz, 1H), 2.12 (d, *J* = 12.5 Hz, 1H), 1.96–1.83 (m, 2H), 1.77 (d, *J* = 13.3 Hz, 1H), 1.55 (d, *J* = 12.4 Hz, 2H). ^1^H NMR showed two conformers in a 70:30 ratio which were further confirmed by low-temperature NMR. ^13^C NMR (201 MHz, CD_2_Cl_2_): δ 166.6, 166.3, 165.6, 164.9, 164.8, 164.7, 162.3, 162.1, 158.8, 158.6, 157.0, 156.6, 156.5, 148.8, 146.0, 144.5, 142.7, 142.6, 142.3, 136.8, 134.8, 134.6, 131.2, 130.8, 130.5, 124.5, 119.6, 114.5, 113.8, 113.7, 112.1, 106.6, 106.4, 101.3, 101.0, 64.3, 58.4, 47.9, 47.2, 43.7, 43.4, 42.8, 41.8, 39.2, 30.0, 23.9, 23.6, 23.4, 22.5. ^13^C NMR showed two conformers in a 70:30 ratio which were further confirmed by low-temperature NMR. **HRMS (ESI)** *m*/*z*: [M + H]+ calcd. for C_32_H_29_N_7_O_11_S, 720.1717; found, 720.1715.

#### Synthesis of *tert*-Butyl 4-(7-methoxy-2-oxo-2*H*-chromene-3-carbonyl)­piperazine-1-carboxylate (**Coum**)

In a 5 mL MW vial, 1-Boc-piperazine (33.5 mg, 0.18 mmol, 1.2 equiv) was dissolved in DMF (500 μL) and basified by the addition of DIPEA (18 μL, 0.1 mmol, 0.7 equiv). The solution was stirred at room temperature for 10 min. A 10 mL round-bottom flask was charged with **2** (33 mg, 0.15 mmol, 1 equiv), HATU (68 mg, 0.18 mmol, 1.2 equiv) and DIPEA (15 μL, 0.08 mmol, 0.5 equiv) in DMF (500 μL) , followed by the addition of the amine solution from the MW vial, and the reaction was left stirring at ambient temperature for 20 min until completion, as indicated by LC–MS. Solvent was then evaporated, and the crude was purified by reversed-phase column chromatography (C18 silica 6 g, 0–100% MeCN/H_2_O) to afford **Coum** (eluted at 70% MeCN) as a white solid (51.5 mg, 88% yield). ^1^H NMR (600 MHz, CDCl_3_): δ 7.89 (s, 1H), 7.42 (d, *J* = 8.7 Hz, 1H), 6.87 (dd, *J* = 8.6, 2.4 Hz, 1H), 6.80 (d, *J* = 2.4 Hz, 1H), 3.87 (s, 3H), 3.70 (t, *J* = 4.9 Hz, 2H), 3.49 (dt, *J* = 17.0, 5.5 Hz, 4H), 3.34 (t, *J* = 5.2 Hz, 2H), 1.44 (s, 9H). ^13^C NMR (151 MHz, CDCl_3_): δ 185.3, 185.1, 179.5, 177.5, 175.7, 165.4, 150.9, 142.1, 134.6, 133.1, 121.8, 101.5, 77.1, 68.3, 65.0, 64.3, 63.4, 49.5. LC–MS (ESI) *m*/*z*: [M + Na]^+^ calcd. for C_20_H_24_N_2_O_6_, 411.1526; found, 411.1525.

### Optical Spectroscopy

Absorption spectra were measured using a spectrophotometer (LAMBDA 950, PerkinElmer). Steady-state emission spectra and excitation spectra were measured with a spectrofluorometer (FLS1000, Edinburgh Instrument) and are corrected using the emission correction files provided by the manufacturer. All absorption and emission spectra were recorded using 10 μM solutions prepared from 10 mM DMSO stock solutions of compounds **Q-amine**, **Coum** and **FRET-Dyad** in 1 cm path length quartz fluorescence cuvette at room temperature. The fluorescence quantum yields were calculated using the relative method, using 9,10-diphenylanthracene in cyclohexane (Φ_F_ = 0.955) as a standard following the IUPAC standard methodology.^39^

### General Methods for Theoretical Calculations

Gaussian 16C01 ^40^ was employed for the density functional theory (DFT) and time-dependent density functional theory (TDDFT) calculations. The geometrical parameters for the ground state (S_0_) were determined via DFT, employing the PBE0 ^41^ and the 6-311+G­(d,p) basis set. Solvent effects were considered by including the solvation model based on density (SMD).^42^ The absolute nature of the energetic minimum was established by the absence of a negative frequency in the vibrational analysis (Table S3). Energy parameters were calculated as vertical electronic excitations from the S_0_ minima structure using the linear response (LR) approach and TDDFT.^43^ These calculations were carried out for the first fifteen excited states at the SMD (water)/PBE0/Def2TZVPP level. The atomic coordinates can be found in the Supporting Information.

Molecular Operating Environment (MOE)^44^ was used to perform molecular dynamics (MD) simulation. Simulation was performed using the MMFF94x force field.^45^ Water (982 molecules) was added as explicit solvent at neutral pH in a spherical cell of 35 Å diameter. The Nosé–Hoover–Andersen (NHA) equations of motion were used.^46^ The DFT minimized **FRET-Dyad** was first subjected to energy minimization within the solvent sphere in an unconstrained environment and under the previously mentioned constraints. Then, the system was preequilibrated for 100 ps at 300 K. Finally, the system was equilibrated at 300 K for 50 ns, with an integration step of 2 fs. The dynamic summary of **FRET-Dyad** is presented in Table S2 and illustrated in Figure S1.

### Plate Reader Fluorescence Measurements

Plate reader measurements were performed on a SpectraMax iD5 microplate reader (Molecular Devices). Kinetic measurements were performed with excitation at 360 nm and emission at 410 nm (gain: medium, integration time: 100 ms; read height: 1.0 mm). Emission spectra were recorded using excitation at 340 nm and emission from 380 to 600 nm (gain: medium, integration time: 100 ms; read height: 1.0 mm). Data were normalized to the plate’s background fluorescence and analyzed in Origin.^47^ Detailed experimental procedures about each experiment can be found in the Supporting Information.

## Results and Discussion

### Design, Synthesis and Characterization of Dyad

We recently reported a structure-reactivity study of sulfone-based-(hetero)­arenes that undergo S_N_Ar reaction with cysteine.^20^ Despite the valuable information gained from the reactivity and stability assays, the workflow and data analysis were quite laborious as all measurements were performed in HPLC/MS. To streamline this process, we aimed to develop a high-throughput platform for evaluating warhead reactivity and stability using a FRET-based system. A sulfone-based cyanopyridine warhead was employed as the molecular bridge between the donor and the acceptor via different linkers. Among the numerous small-molecule FRET pairs reported in the literature,^48^ those containing coumarins as FRET-donor in their scaffold are the most extensively explored. The widespread use of coumarins in this context rests on their favorable photophysical properties and their versatility as sensors for different analytes and biologically active molecules.^49^ We selected 7-methoxy-coumarin as a donor fluorophore for our system because of its straightforward synthesis and high fluorescence quantum yield in aqueous environments.^50^ To achieve optimal FRET-efficiency, a dinitrophenyl dark quencher was employed as the acceptor due to its excellent spectral overlap with 7-methoxy-coumarin.^48^ A piperidine linker was incorporated to promote optimal orbital alignment and minimize possible reorientation events that could arise from using highly flexible linkers. The reaction of the dyad with a nucleophile releases the coumarin donor, leading to a turn-on fluorescence response (Figure 2).

**Figure 2:**
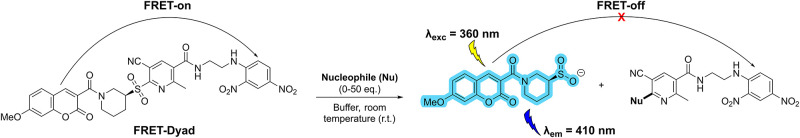
Response mechanism of **FRET-Dyad** toward nucleophiles.

The synthetic route for the **FRET-Dyad** is depicted in Scheme 1, and detailed synthetic protocols and characterization data can be found in the Supporting Information. Briefly, coumarin acid **2** was obtained via a Knoevenagel condensation between diethyl malonate and 2-hydroxy-4-methoxybenzaldehyde, followed by ester hydrolysis under basic conditions. The corresponding quencher amine (**Q-amine**) was prepared through an S_N_Ar reaction between 1-fluoro-2,4-dinitrobenzene with *N*-Boc ethylenediamine, and subsequent acidic Boc deprotection. The cyanopyridine warhead **6** was synthesized via a Mitsunobu reaction between thiol **5** and (*S*)-1-Boc-3-hydroxypiperidine. Subsequent hydrolysis of the ethyl ester **6**, and amide coupling with the **Q-amine**, afforded intermediate **7**, which was then subjected to TFA-mediated Boc-deprotection and amide coupling with coumarin acid **2**. Oxidation of the sulfide group with mCPBA furnished the final **FRET-Dyad** in 73% yield over 3 steps. Since **FRET-Dyad** is linked to the cyanopyridine warhead via a piperidine linker, a coumarin-derivative (**Coum**) was also synthesized as a model for optical measurements. **Coum** was prepared via amide coupling between 1-Boc-piperazine and coumarin acid **2**.

**Scheme 1:**
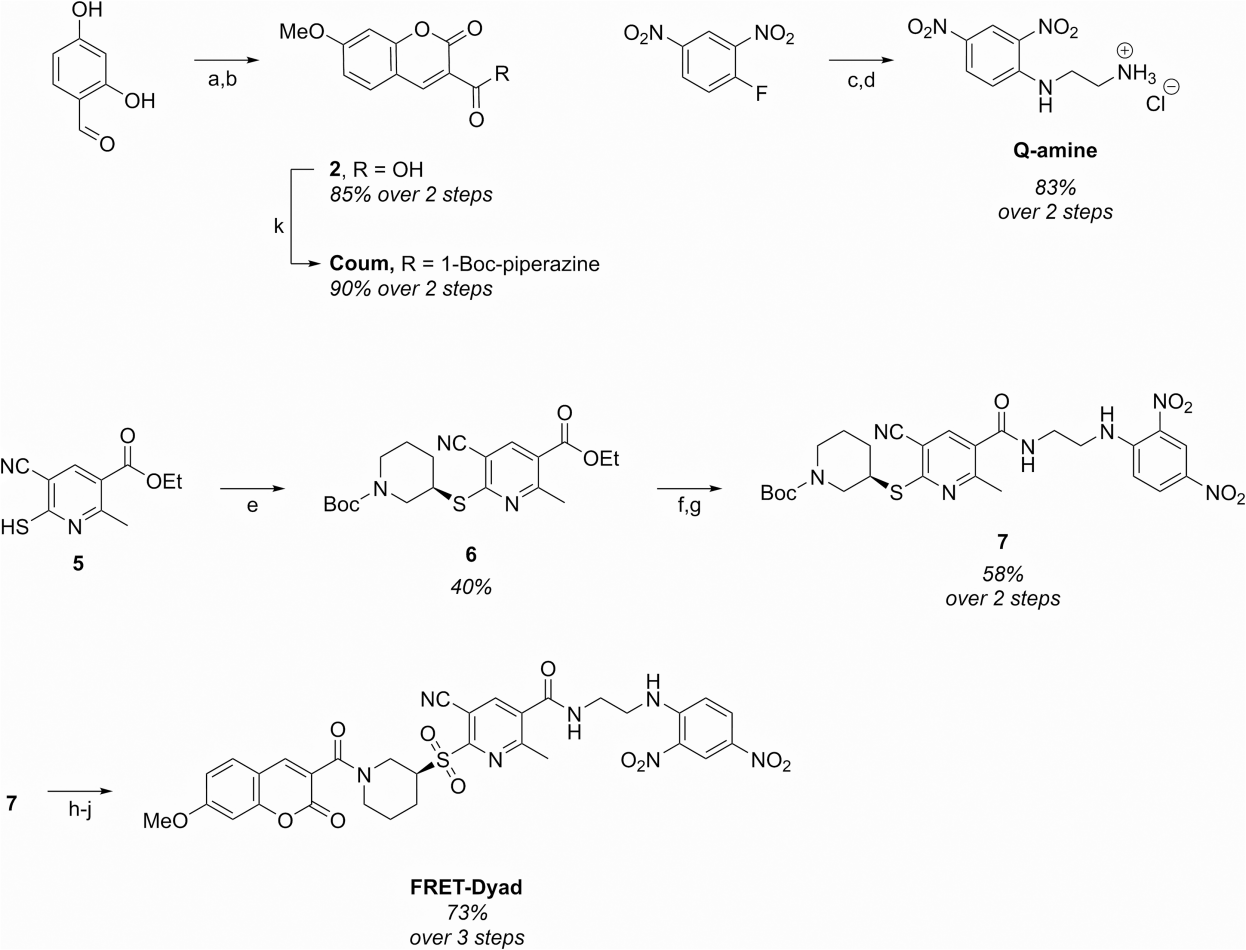
Synthesis of **FRET-Dyad** ^a^

a Reagents and conditions: (a): Diethyl malonate (1 equiv), piperidine (cat.), room temperature (rt), 30 min; (b) NaOH (10%), EtOH, reflux, 15 min; (c) *N*-Boc ethylenediamine (1.1 equiv), TEA (2 equiv), THF, rt , 2 h; (d) HCl 4 M in 1,4-dioxane (10 equiv), MeOH, 0 °C to rt, 16 h; (e) (*S*)-1-Boc-3-hydroxypiperidine (1.50 equiv), CMBP (1.5 equiv), toluene, 110 °C, 16 h; (f) LiOH (7 equiv), EtOH, 0 °C to rt, 3 h; (g) NHS (1.3 equiv), EDCI (1.3 equiv), DMF, rt, 2 h, then **Q-amine** (1 equiv), DIPEA (1.1 equiv), DMF, rt, 1 h; (h) TFA (26 equiv), DCM, 0 °C to rt, 6 h; (i) HATU (1.2 equiv), DIPEA (2.4 equiv), **2** (1 equiv), DMF, rt, 1 h; (j) mCPBA (5 equiv), CHCl_3_, 0 °C to rt, 5 h; (k) 2 (1 equiv), HATU (1.2 equiv), DIPEA (1.2 equiv), DMF; 1-Boc-piperazine (1.2 equiv), rt, 20 min.

### Spectroscopic Properties of FRET-Dyad

We first investigated the spectroscopic properties of **Coum**, **Q-amine**, and **FRET-Dyad** in aqueous solutions using UV–vis absorption and fluorescence spectroscopy. The photophysical properties of all compounds were examined in water and TRIS buffer (pH 7.4), using 10% DMSO as co-solvent. The spectra in water are shown in Figure 3a, and the key photophysical data are summarized in Tables 1 and S1. The absorption band of **Coum** is relatively broad with a maximum at 340 nm and a molar absorption coefficient ε of 21,700 M^–1^ cm^–1^. The emission spectrum of **Coum** displays a mirror image relationship to its absorption, with a maximum centered around 402 nm. The low fluorescence quantum yield (Φ_F_ = 0.0222) could be attributed to the substitution pattern of **Coum**, as a reduced emission quantum yield through a tertiary amide substituent in the 3-position has been described previously.^51^ Regarding **Q-amine**, the absorption band is composed of two distinct electronic transitions: a primary absorption band centered at 352 nm and a secondary transition at 416 nm, appearing as a shoulder, a feature consistent with previous reports.^52^ The combination of these spectral features results in a substantial spectral overlap between the emission of **Coum** and the absorption of **Q-amine**, which is evident both visually (Figure 3a) and quantitatively from the calculated overlap integral (2.5 × 10^14^ nm^4^ M^–1^ cm^–1^). Additionally, solvent effects were minimal: switching from water to TRIS buffer (pH 7.4, 10% DMSO as co-solvent) slightly decreased fluorescence quantum yields but did not change absorption or emission maxima (Table S1).

**Figure 3:**
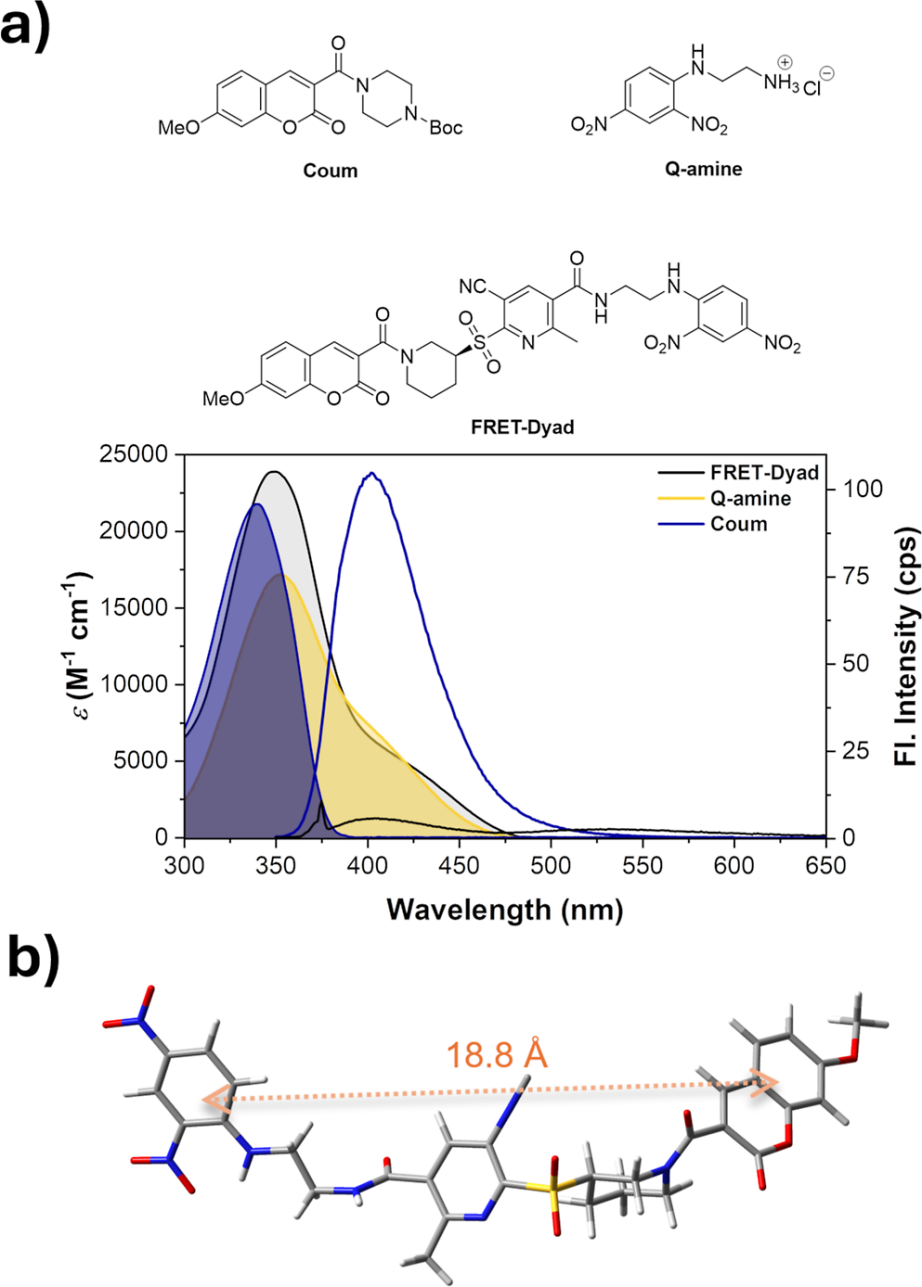
Spectroscopic properties and optimized geometry of the FRET-Dyad. (a) Absorption spectra (filled areas) of **Coum** (blue), **Q-amine** (yellow), and **FRET-Dyad** (light grey), and emission spectra (solid lines, excitation at 340 nm) of **Coum** (blue) and **FRET-Dyad** (black). Samples were prepared in an air-equilibrated 10% DMSO-water solution. The peak centered around 360 nm in the emission spectrum of the dyad corresponds to Raman scattering and not fluorescence. (b) Optimized ground-state geometry of **FRET-Dyad** calculated at the SMD (water)/PBE0/6-311+G­(d,p) level of theory. The orange arrow connects the donor and acceptor centers of mass, and the interchromophore distance is indicated.

**Table 1:** Photophysical Properties of all Compounds in Water (10% DMSO)

compound	λ_abs_ (nm)^a^	ε (M-^1^ cm^–1^)^b^	λ_em_ (nm)^c^	Φ_F_ ^d^
**Coum**	340	21,700	402	0.0222
**Q-amine**	416,352	5500 17,200	nd	nd
**FRET-Dyad**	416,350	5300 23,900	402	0.0017

a Absorption wavelength.

b Molar absorption coefficient.

c Emission maxima upon excitation at 340 nm.

d Fluorescence quantum yield; measured using as standards 1,9-diphenylantracene in cyclohexane.

Based on the experimental data in water, the Förster radius was calculated to be 21.3 Å according to FRET theory. Considering the interchromophore distance obtained from geometry optimization by DFT (18.8 Å, Figure 3b), the theoretical FRET efficiency was estimated to 68%. However, the experimentally determined value of 92% suggests a more efficient process. This discrepancy may arise from several factors contributing to the higher experimentally observed FRET efficiency, such as the orientation factor and the interchromophore distance.^53^ In relation to the orientation factor (κ^2^), this was assumed to be 2/3, which may lead to underestimation of the Förster radius. The presence of conformational isomers, as observed by NMR (Figures S24–S26), supports this notion and suggests that conformational flexibility could contribute to deviations in the calculated FRET efficiency. Secondly, the calculated donor–acceptor (D–A) distance represents the maximal donor-to-acceptor distance in the lowest energy conformation. Nevertheless, the **FRET-Dyad** may adopt multiple conformations in solution, in which the effective interchromophore distance may be reduced. To gain insights into this conformational behavior, molecular dynamics (MD) simulations were additionally performed. Briefly, **FRET-Dyad** was solvated with explicit water in a spherical cell, and the system was equilibrated for 50 ns at 300 K. The analysis of the MD trajectory revealed an average D–A distance of 15.9 ± 1.3 Å (Figure S1 and Table S2), which is shorter than the distance previously estimated by DFT calculations. Assuming once again the orientation factor (κ^2^) to be 2/3 and the distance for each conformation during the MD trajectory, the FRET efficiency was estimated to be 85% on average (79–91%), which is more consistent with the experimental findings. While these simulations provide insight into conformational flexibility and donor–acceptor distances, a full assessment of the distribution and dynamics of the dyad’s orientation on the average FRET efficiency would require a more extensive computational analysis. Such an analysis may explain the observed discrepancy between theoretical and experimental findings; however, it is beyond the scope of the present study. The present computational analysis nevertheless provides a qualitative structural basis for interpreting the observed fluorescence behavior.

### Theoretical Calculations

To support the experimental findings, additional theoretical calculations were performed to examine the optical properties of the **FRET-Dyad**. Analysis of the calculated absorption spectra (Table 2) reveals that the absorption spectrum of the **FRET-Dyad** in the visible region is mainly described by three electronic transitions: S_0_ → S_1_ and S_0_ → S_3_, related to the **Q-amine** moiety, and S_0_ → S_5_, associated exclusively to **Coum**. The Frontier molecular orbitals (FMOs) participating in these electronic transitions are represented in Figure 4 and support this assignment. All these transitions are characterized by a π–π* character, although their electronic nature differs slightly. The transitions related to **Q-amine** exhibit pronounced changes in electron density upon excitation, indicating an intramolecular charge transfer character.^52^ In contrast, the electron density in the FMOs involved in the S_0_ → S_5_ transition is largely localized within the same region of the molecule, which is typically associated with a locally excited state. Notably, no electronic mixing between the chromophores is observed, i.e., the individual electronic transitions are localized exclusively on one of the chromophores within the dyad. The absence of electronic mixing is also observed in the composition of the transitions (Table 2). This notion is also supported by the results from the MD simulation: the average D–A distance in the **FRET-Dyad** over the trajectory suggests that the compound predominantly adopts an extended conformation in solution, comparable to that determined by DFT optimization. The calculated geometries show that the donor and acceptor chromophores remain spatially separated and do not exhibit significant orbital overlap. Such configurations are likely unfavorable for ground-state electronic coupling or through-bond charge transfer between the two chromophores, which could otherwise perturb their individual electronic properties.

**Table 2:** Calculated Electronic and Photophysical Data for the **FRET-Dyad**

electronic transition	*f* ^a^	assignation^b^	FMO composition (contribution^c^)	*E*_theo_ (eV)^d^ [λ (nm)]	*E*_exp_ (eV)^e^[λ (nm)]
S_0_ → S_1_	0.264	**Q-amine**	HOMO-1 → LUMO (99)	2.86 [433.53]	2.98 [416]
S_0_ → S_3_	0.610	**Q-amine**	HOMO-1 → LUMO+1 (98)	3.31 [374.41]	3.53 [351]
S_0_ → S_5_	0.881	**Coum**	HOMO → LUMO+3 (93)	3.56 [348.79]	3.67 [338]

a Oscillator strength.

b Based on NTOs visual inspection (see figure below).

c Percentage contribution approximated by 2ci 2 × 100%.

d Absorption energies calculated at the SMD (water)/PBE0/6-311+G­(d,p) level of theory.

e Experimental absorption energies in air-equilibrated aqueous solution of the model monomers composing **FRET-Dyad**. Please note that the absorption spectra of **Q-amine** were analyzed by mathematical deconvolution to identify the different bands composing it.

**Figure 4:**
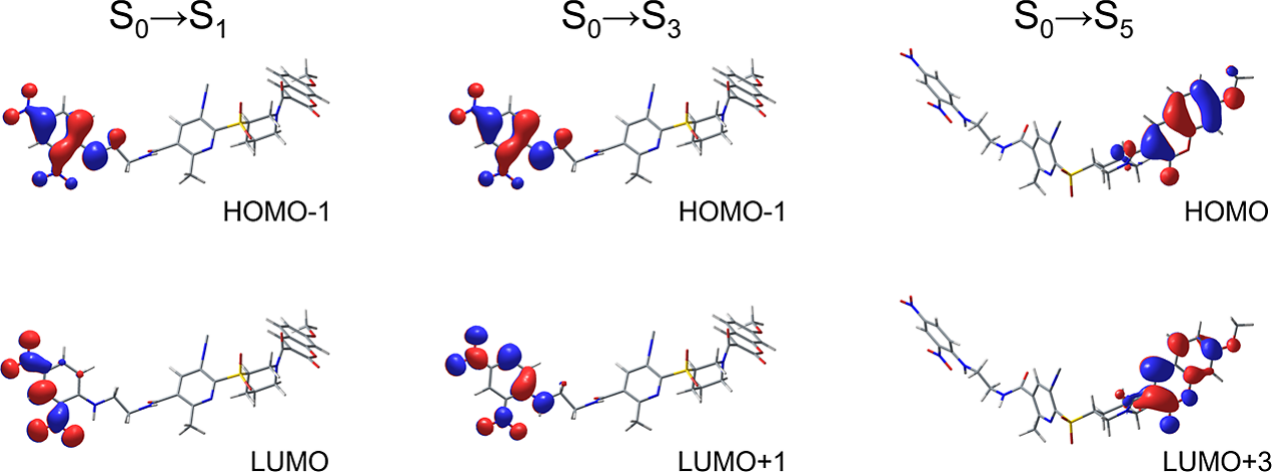
Frontier molecular orbitals (FMOs) participating in S_0_ → S_1_, S_0_ → S_3_, and S_0_ → S_5_ transitions in **FRET-Dyad** (isosurface: 0.03 e/bohr^3^).

Despite minor discrepancies between the calculated and experimental energy values (Table 2), the theoretical model provides a reasonable description of the system, with energy deviations ≤0.25 eV.^54,55^ This good agreement is also illustrated by comparing experimental and calculated absorption spectra of **FRET-Dyad** (Figure S2).

Taken together, these observations are consistent with energy transfer in this system occurring predominantly through the FRET mechanism.

### Fluorescence Response of FRET-Dyad to Nucleophiles

The fluorescence response of **FRET-Dyad** toward nucleophiles was investigated to establish a robust assay platform that allows simultaneous testing of multiple parameters. For that, all assays were performed in either 384- or 96-well plate formats and fluorescence was recorded in a plate reader. The optimal dyad concentration as well as buffer composition were first examined (Figures 5a and S3). To better gauge the intrinsic reactivity, NAC and GSH were used as model thiols. GSH is the most abundant (1–10 mM) intracellular nonprotein thiol,^56^ acting as an antioxidant and redox regulator but can also quench electrophiles,^1^ and is therefore commonly employed as a model thiol nucleophile for warhead reactivity assays.

**Figure 5:**
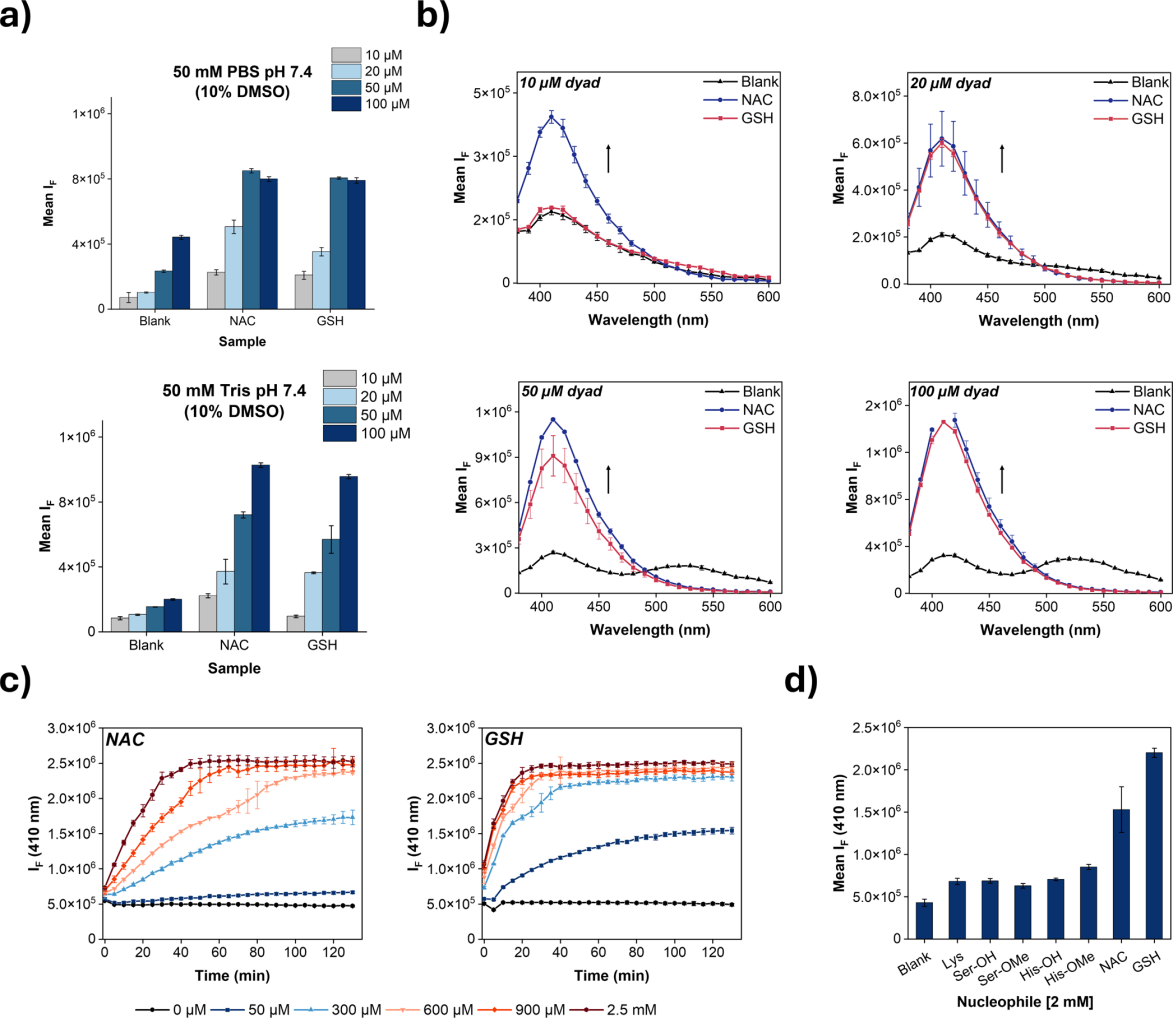
Fluorescence response of the **FRET-Dyad** to nucleophiles. (a) Changes in fluorescence emission at 410 nm of **FRET-Dyad** (10–100 μM) after incubation with NAC or GSH (50 equiv) for 1 h at rt in 50 mM PBS or 50 mM TRIS buffer pH 7.4 containing 10% DMSO as co-solvent. Fluorescence is presented as relative fluorescence units (RFU). Data are presented as the mean ± SD (*n* = 3). (b) Fluorescence emission spectra of **FRET-Dyad** (10–100 μM) in the absence and presence of 50 equiv NAC or GSH in 50 mM TRIS buffer pH 7.4 (10% DMSO as co-solvent) (λ_ex_ = 360 nm, λ_em_ = 380–600 nm incubation time = 1 h). Data are presented as the mean ± SD (*n* = 3). (c) Plot of fluorescence intensity change of **FRET-Dyad** (50 μM) as a function of incubation time with different NAC and GSH concentrations (TRIS or PBS buffer pH 7.4, 10% DMSO as co-solvent, rt, 2 h, λ_exc_ = 360 nm, λ_em_ = 410 nm). Data are presented as the mean ± SD (*n* = 4). (d) Fluorescence responses of **FRET-Dyad** (50 μM) after incubation for 4 h at rt with different nucleophiles (50 equiv λ_exc_ = 360 nm, λ_em_ = 410 nm), 50 mM PBS buffer, pH 8.3 10% DMSO as co-solvent. Data are presented as the mean ± SD (*n* = 4).

Different concentrations of **FRET-Dyad** (10–100 μM) were incubated with either NAC or GSH as model thiols for 1 h in different buffers, using 10% DMSO as co-solvent. To better quantify the reaction rates, the nucleophile was used in excess (50 equiv for each dyad concentration, pseudo-first-order conditions). To ensure adequate solubility of the dyad under these conditions, all reagents were first mixed in Eppendorf tubes, and 20 μL of each reaction mixture were transferred into a 384-well plate in triplicates. For all samples, the fluorescence intensity at 410 nm increased proportionally with probe concentration (3- to 4-fold difference) with the higher intensity observed at 100 μM, albeit with a saturation effect at 410 nm. To verify that the increase in fluorescence originates from the action of the nucleophiles and not due to aggregation-induced emission of the coumarin-donor, an aliquot of the reaction mixture was injected in the LC–MS, verifying the formation of the arylated-thiol conjugate and the release of the coumarin (Figures S4 and S5). Although the dyad exhibits negligible fluorescence in all blank samples, a secondary, broader emission band centered at 530 nm was detected for the 50 μM and 100 μM samples (Figure 5b), likely associated with aggregate formation at higher concentrations. Nevertheless, a probe concentration of 50 μM was selected for subsequent assays, as it provided a sufficient assay window. Among the tested buffers, we chose TRIS for our further investigations due to the discrepancies observed in the different blank samples in PBS buffer (Figures 5a, S3 and S6).

With an established assay in hand, we then performed titration experiments of the dyad toward NAC and GSH at low analyte concentrations. The FRET-based workflow was readily implemented on an automated liquid-handling platform (Opentrons OT-2), enabling consistent reagent dispensing and minimizing variability across 96-well plates. The automation improved assay reproducibility (RSD <5%) and throughput. The dependence of emission of **Coum** with time was monitored at 410 nm over a period of 2 h. As illustrated in Figure 5c, fluorescence intensity increased significantly upon addition and reached a plateau after 1 h of incubation with LC–MS analysis confirming complete conversion (Figure S7a). Reaction rates increased linearly with nucleophile concentration (Figures 5c and S7b), and second-order rate constants indicated that GSH (*k*_2_ ≈ 0.62 M^–1^ s^–1^) reacted nearly twice as fast as NAC (*k*_2_ ≈ 0.37 M^–1^ s^–1^) at pH 7.4. The pK_a_ values of the thiol group in NAC and GSH are 9.4 ^57^ and 9.2 ^58^ respectively, which could explain the difference in reactivity. Note that for GSH, PBS buffer seemed to be the optimal choice as the increase in fluorescence in TRIS showed some deviations from the linear range (Figure S8). As expected, the reaction proceeded faster at higher pH, with the fluorescent signal showing a linear increase as a function of pH (Figure S9a). To confirm that the fluorescence increase is attributed to the higher effective equilibrium concentration of the thiolate anion and not to hydrolysis products, a pH stability assay was also performed. For this, **FRET-Dyad** was dissolved in different buffers with pH ranging from 7.4 to 10, and the fluorescence intensity at 410 nm was monitored over time. No hydrolysis of the dyad was observed in any of the buffers, as evidenced by the absence of increase in fluorescence intensity (Figure S9b).

Because the maximum fluorescence signal was lower than that of the isolated coumarin donor, we evaluated whether the released quencher caused intermolecular FRET. Indeed, titration of **Coum** with increasing concentrations of **Q-amine** showed a concentration-dependent decrease in fluorescence (Figure S10), explaining the reduced final signal.

Finally, we investigated the dyad’s chemoselectivity profile toward different amino acids, including *N*_a_-acetyl lysine, *N*-Boc serine, and *N*-acetyl histidine. These residues are often found in the active protein sites and having perturbed p*K*_a_ values, as the respective protein environment can have a large impact on their protonation state.^1,21^ For that reason, the reactivity was assessed at higher pH (50 mM PBS buffer, pH 8.3, 10% DMSO). The nucleophile was again used in excess and as a control, NAC and GSH were tested in the same buffer composition. As shown in Figure 5d, no significant increase in fluorescence was observed for any of the amino acid derivatives except for the two cysteine nucleophiles under these conditions. The minimal difference in fluorescence readout between the blank and the analytes corresponds to partial hydrolysis of the dyad after 4 h, as validated by LC–MS. This result was more pronounced when the assay was performed at 37 °C (Figure S11), therefore highlighting the selectivity of our S_N_Ar-warhead toward cysteine nucleophiles.

## Conclusions

We developed a FRET-based fluorescence assay for evaluating covalent warhead reactivity. This platform allows parallel screening of nucleophiles and assay conditions, such as buffer composition, in a 96-well plate format, providing quantitative kinetic information while reducing the experimental workload. Using a sulfone-based S_N_Ar electrophile as a model warhead, the assay demonstrated excellent selectivity for thiols over other nucleophiles, supporting its suitability for high-throughput profiling of electrophile reactivity under physiologically relevant conditions. Because the electrophile is incorporated into a modular donor-warhead-quencher design, this approach can be readily extended to other covalent warheads, including acrylamides, and heteroaryl sulfones. Although intrinsic warhead reactivity in solution does not fully translate to warhead reactivity in proteins, this approach nonetheless provides a practical tool for profiling electrophile reactivity by enabling direct assessment of covalent reaction kinetics. Future studies will focus on applying this framework to systematically assess reactivity across diverse electrophilic warhead classes. Beyond chemical biology applications, such a platform may also support early-stage prioritization of covalent warheads in drug discovery.

## Supplementary Material





## Notes

Details about supporting figures, synthetic schemes, fluorescence experiments, plate reader methods and characterization data (PDF)
(XYZ)

## Abbreviations

(AcOH): acetic acid
(MeCN): acetonitrile
CMBP: ((cyanomethylene)­tributylphosphorane)
(DCM): dichloromethane
(DIPEA): *N*,*N*′-diisopropylethylamine
(DMF): *N*,*N*′-dimethylformamide
(EtOAc): ethyl acetate
(DMSO): dimethyl sulfoxide
(EDC): 1-ethyl-3-(3-dimethylaminopropyl) carbodiimide
(HATU): 1-[*bis*(dimethylamino)­methylene]-1*H*-1,2,3- triazolo­[4,5-*b*]­pyridinium3-oxidhexafluorophosphate
(MeOH): methanol
(NHS): *N*-hydroxysuccinimide
(THF): tetrahydrofuran
(TFA): trifluoroacetic acid
