# Supplementary material for: A FRET-Based Assay for Assessing Covalent Warhead Reactivity
Source: ACS Omega. 2026 Jun 24;11(26):38475–86. doi: 10.1021/acsomega.5c12902 (PMC13347334; doi:10.1021/acsomega.5c12902)
Supplement: Supplementary file 1 [file ao5c12902_si_001.pdf]

Supporting Information

A FRET-based assay for assessing covalent warhead reactivity

*Anna P. Valaka[1], Carlos Benitez-Martin[1], Joakim Andréasson[2] and Morten Grøtli[1]**

[1]Department of Chemistry and Molecular Biology, University of Gothenburg, 405 30, Gothenburg, Sweden.[2]Department of Chemistry and Chemical Engineering, Chalmers University of Technology, 412 96, Gothenburg, Sweden.

***Corresponding author.**

Table of contents

Experimental Procedures 2
Equations 2
Plate Reader Fluorescence Measurements 2
Effect of dyad concentration and buffer composition on fluorescence readout 2
Effect of nucleophile concentration on the rate of reaction and hydrolytic stability 2
Effect of pH on reaction kinetics and hydrolytic stability 3
Investigation of intramolecular quenching 3
Selectivity 3
Supplementary Figures and Tables 4
NMR Spectra 15
References 32

Experimental Procedures

Equations

Equation 1 - Stern-Volmer equation[1]

$$\frac{I_0}{I} = 1 + K_{SV}\ [Q]$$

where I_0 and I are, respectively, the intensity in the absence and presence of a quencher, K_{SV} is the Stern-Volmer constant and [Q] is the quencher concentration.

Equation 2 - Second rate constant[2]

$$k_{obs} = k_2[Nu]$$

where k_2 is the pseudo-first order reaction rate constant and [Nu] the nucleophile concentration.

Plate Reader Fluorescence Measurements

Effect of dyad concentration and buffer composition on fluorescence readout
NAC or GSH was added separately to 0, 10, 20, 50 and 100 µM **FRET-Dyad** in 50 mM PBS pH 7.4, 50 mM TRIS pH 7.4, 50 mM PBS pH 8.3, or 100-mM borate buffer pH 8.3. In all buffer conditions, 10% DMSO was used as a co-solvent. For pseudo-first-order kinetics, 50 eq. of the thiol was used for each dyad concentration (0.5, 1, 2.5 and 5 mM respectively). Stock solutions of GSH and NAC in each buffer were prepared fresh at 0.1 M, in the respective buffer immediately before the addition. Reactions were performed in Eppendorf tubes (600 µL total volume) for 1 h with shaking at rt. After 1 h, 20 µL of the reaction mixture were transferred into 384-well plates (Corning Black PS, 3820) in triplicates and emission spectra as well fluorescence intensity (endpoint measurement) were recorded as stated above. In all cases, formation of the arylated product was verified by LC-MS.

Effect of nucleophile concentration on the rate of reaction and hydrolytic stability
FRET-Dyad (50 µM, 5 µL from a 100 µM stock in DMSO) was added separately to 0, 50, 300, 600, 900 and 2500 µM NAC or GSH. For NAC, reactions were performed in 50 mM TRIS buffer pH 7.4, whereas for GSH, reactions were performed in both 50 mM TRIS and 50 mM PBS buffers at pH 7.4. Both buffers contained 10% DMSO as a co-solvent. Reactions were run in a total reaction volume of 100 µL in 96-well plates (Greiner, 655076) in quadruplicates. Apart from the dyad, all

reagents were added via a liquid handler (OT-2, Opentrons). Stock concentrations of the nucleophiles were prepared fresh at 1 and 5 mM in the respective reaction buffer. Fluorescence intensity measurements were immediately acquired at 410 nm at rt every 5 min for 2 h. Normalized fluorescence intensities of each sample were plotted as a function of time (min), allowing extraction of the pseudo-first order reaction rate constant (k_{obs}).[2] Second order rate constants (k_2) were back calculated from the ratio of the pseudo-first counterparts and the concentration of thiol in solution, and fitted to a linear fit in OriginPro.[3]

Effect of pH on reaction kinetics and hydrolytic stability

FRET-Dyad (50 µM, 5 µL from a 100 µM stock in DMSO) was added separately to 2.5 mM GSH in 50 mM PBS buffer at pH 6, 7.4, 8.3, and 10.5. Stock solutions of GSH were prepared fresh at 100 mM in the respective reaction buffer. Reactions were run in a total reaction volume of 100 µL in 96-well plates (Greiner, #655076) in quadruplicates. Fluorescence intensity measurements were immediately acquired at 410 nm at rt every 5 min for 2 h. The mean fluorescence intensities of each sample were plotted as a function of time (min). The assessment of hydrolytic stability was conducted at pH 7.4, 8.3 and 10.5 in a similar manner in the absence of thiol nucleophiles.

Investigation of intramolecular quenching

In a 1.5 mL Eppendorf tube, **Coum** (50 µM, 5 µL from a 6 mM stock in DMSO) was added separately to 0, 5, 10, 15, 30 and 50 µM of **Q-amine** (1 mM stock in DMSO) in 50 mM TRIS buffer pH 7.4, containing 10% DMSO as co-solvent in a total volume of 600 µL. Samples were vortexed and spun down, and 20 µL of each mixture were transferred into 384-well plates (Corning Black PS, 3820) in triplicates and emission spectra as well as fluorescence intensity were recorded as stated above. The mean intensities of the emission spectra were plotted at the different concentrations of the quencher and the average intensities at 410 nm were fit to the Stern-Volmer equation using least squares regression with *y*-intercept set to 1. The Stern-Volmer constant was derived from the slope of linear fit.[1]

Selectivity

FRET-Dyad (50 µM, 5 µL from a 100 µM stock in DMSO) was added separately to 2.5 mM of amino acid solution in 50 mM PBS buffer pH 8.3 containing 10% DMSO as co-solvent. N_α-acetyl lysine, *N*-Boc-Ser-OH, *N*-Boc-Ser-Ome, *N*-Boc-His-OH and *N*-Boc-His-OMe were used. Reactions were run in a total reaction volume of 100 µL in 96-well plates (Greiner, #655076) in quadruplicates. Apart from the dyad, all reagents were added via a liquid handler (OT-2, Opentrons). Stock concentrations of the nucleophiles were prepared fresh at 1 and 5 mM in 50 mM PBS buffer pH 8.3. Fluorescence intensity measurements were immediately acquired at 410 nm at rt every 15 min for 4 h. The mean fluorescence intensities of each sample were plotted as a function of time (min) and the average fluorescence intensity was plotted for each nucleophile. The assay was also conducted at 37 °C, following the same set-up. In this case, fluorescence intensity at 410 nm was measured before the addition of the analyte and after 4 h of incubation time at 37 °C.

Supplementary Figures and Tables

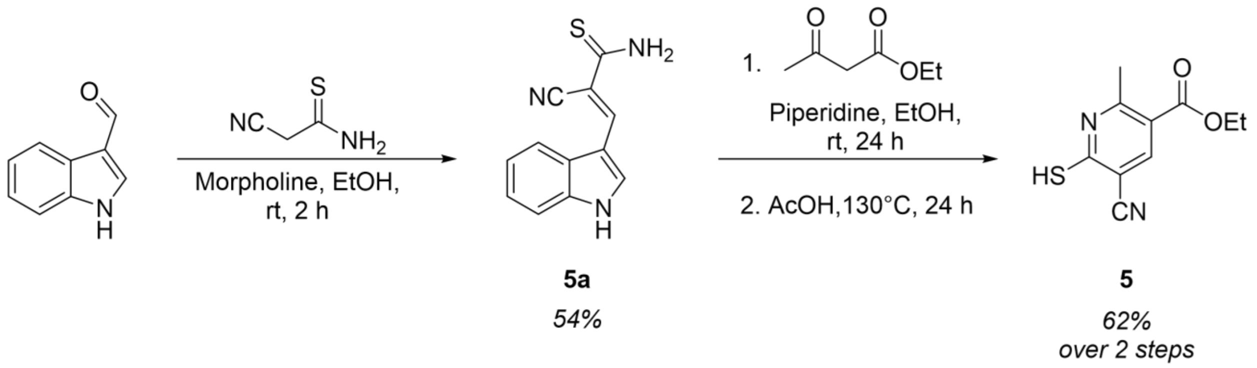

Scheme S1. Synthesis of ethyl 5-cyano-6-mercapto-2-methylnicotinate (**5**) from indole-3-carboxaldehyde and 2-cyanothioacetamide.

Table S1. Photophysical properties of all compounds in TRIS buffer (pH 7.4, 10% DMSO).

Compound	λ_{abs} (nm)[a]	λ_{em} (nm)[b]	Φ_F[c]
**Coum**	339	401	0.0124
**Q-amine**	416<br>353	*nd*	*nd*
**FRET-Dyad**	416<br>350	402	0.0012

[a]Absorption wavelength. [b]Emission maxima upon excitation at 340 nm. [c]Fluorescence quantum yield; measured using 1,9-diphenylantracene in cyclohexane as standard. nd: not detected.

Table S2. Dynamic summary of **FRET-Dyad**.

Dynamics summary	Average (50 ns)	Std. Dev. (50 ns)
Total energy (kcal/mol)	-6564.255	61.453
Potential energy (kcal/mol)	-8674.497	50.017
Kinetic energy (kcal/mol)	2110.242	35.477
Temperature (K)	300.016	5.044
D-A distance (Å)	15.907	1.271

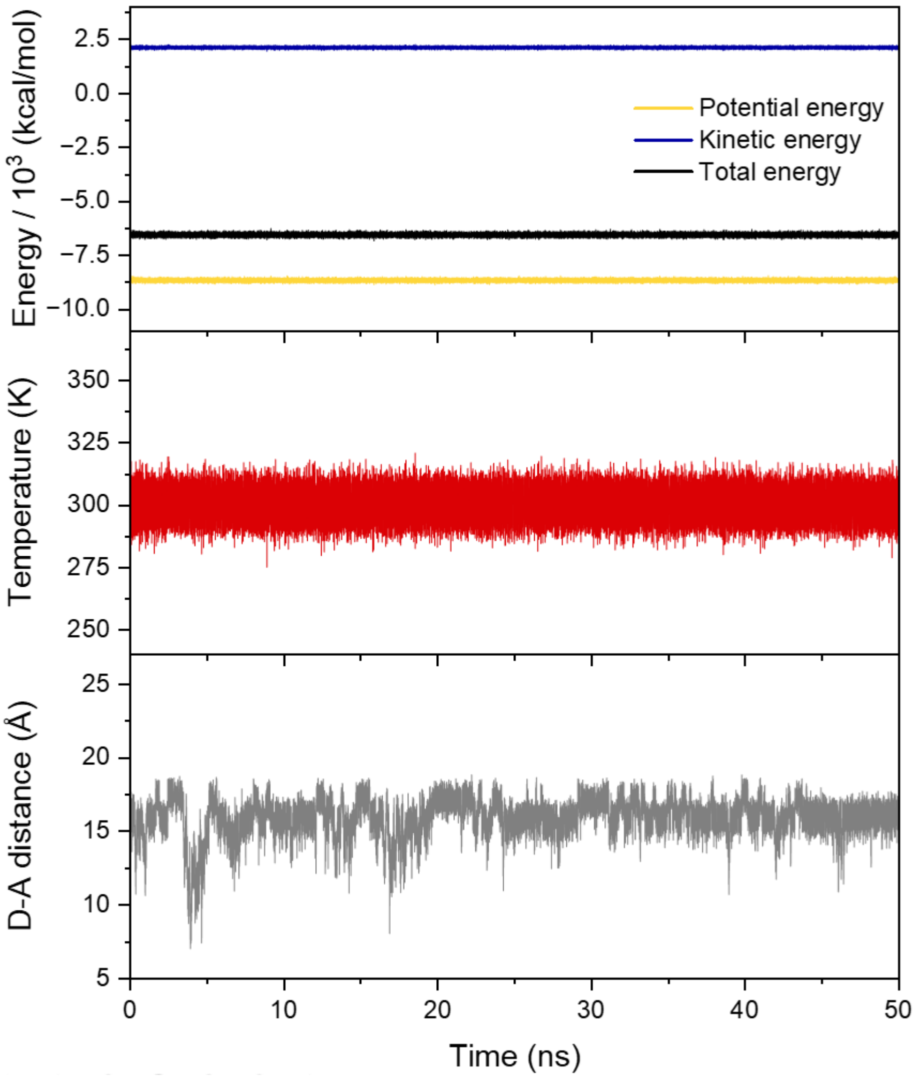

Figure S1. Molecular Dynamics (MD) trajectory analysis of the donor-acceptor distance for 50 ns at 300 K. The **FRET-Dyad** was solvated with explicit water in a spherical cell, and the system was then equilibrated. Energy, temperature, and D-A distance profiles of the 50 ns MD simulation on **FRET-Dyad** are indicated from top to bottom, correspondingly. The average distance of the MD trajectory was calculated to be 15.9 ± 1.3 Å.

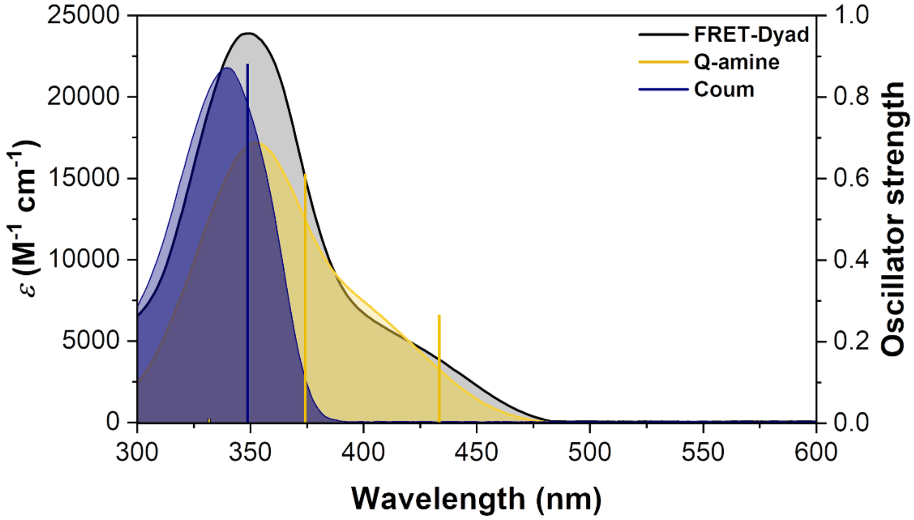

Figure S2. Experimental absorption spectra (filled area) of **Coum** (blue), **Q-amine** (yellow), and **FRET-Dyad** (light grey), and calculated absorption spectra (vertical lines) of **FRET-Dyad** (please note that the different transitions are colored according to their assignment).

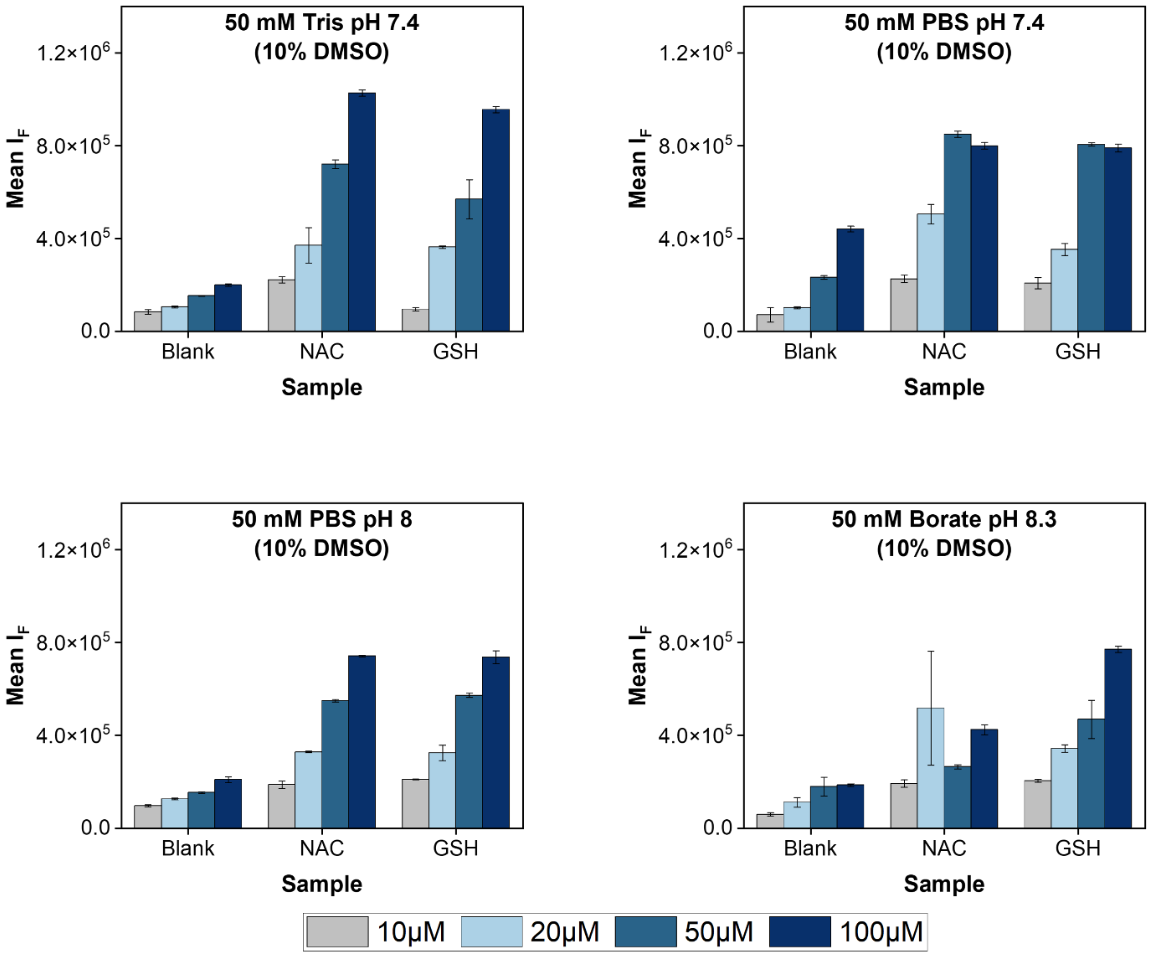

Figure S3. Changes in fluorescence emission at 410 nm of **FRET-Dyad** (10-100 µM) after incubation with NAC or GSH (50 eq.) for 1 h at rt in different buffers. Fluorescence is presented as relative fluorescence units (RFU). Data represent mean ± SD (n = 3).

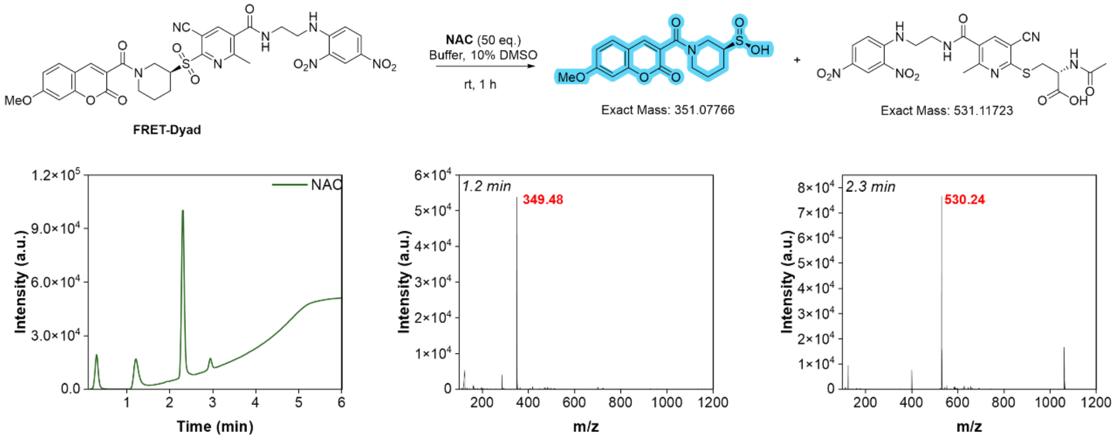

Figure S4. UV spectra (sum of absorption at 254 nm and 360 nm) of the LC-MS analysis of 100 µM of **FRET-Dyad** incubated with 5 mM NAC for 1 h in TRIS buffer pH 7.4 (10% DMSO) at room temperature.

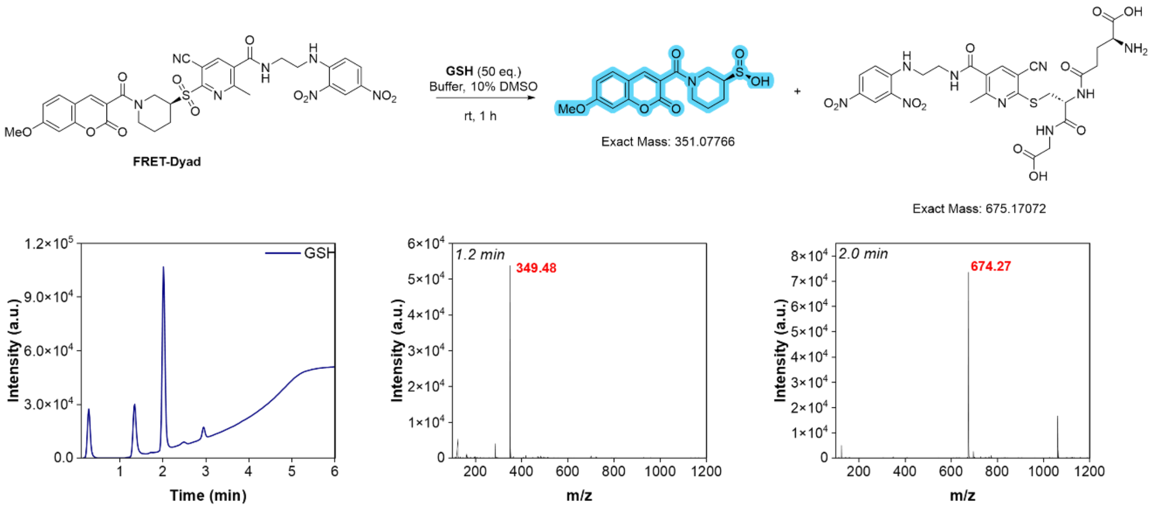

Figure S5. UV spectra (sum of absorption at 254 nm and 360 nm) of the LC-MS analysis of 100 µM of **FRET-Dyad** incubated with 5 mM GSH for 1 h in TRIS buffer pH 7.4 (10% DMSO) at room temperature.

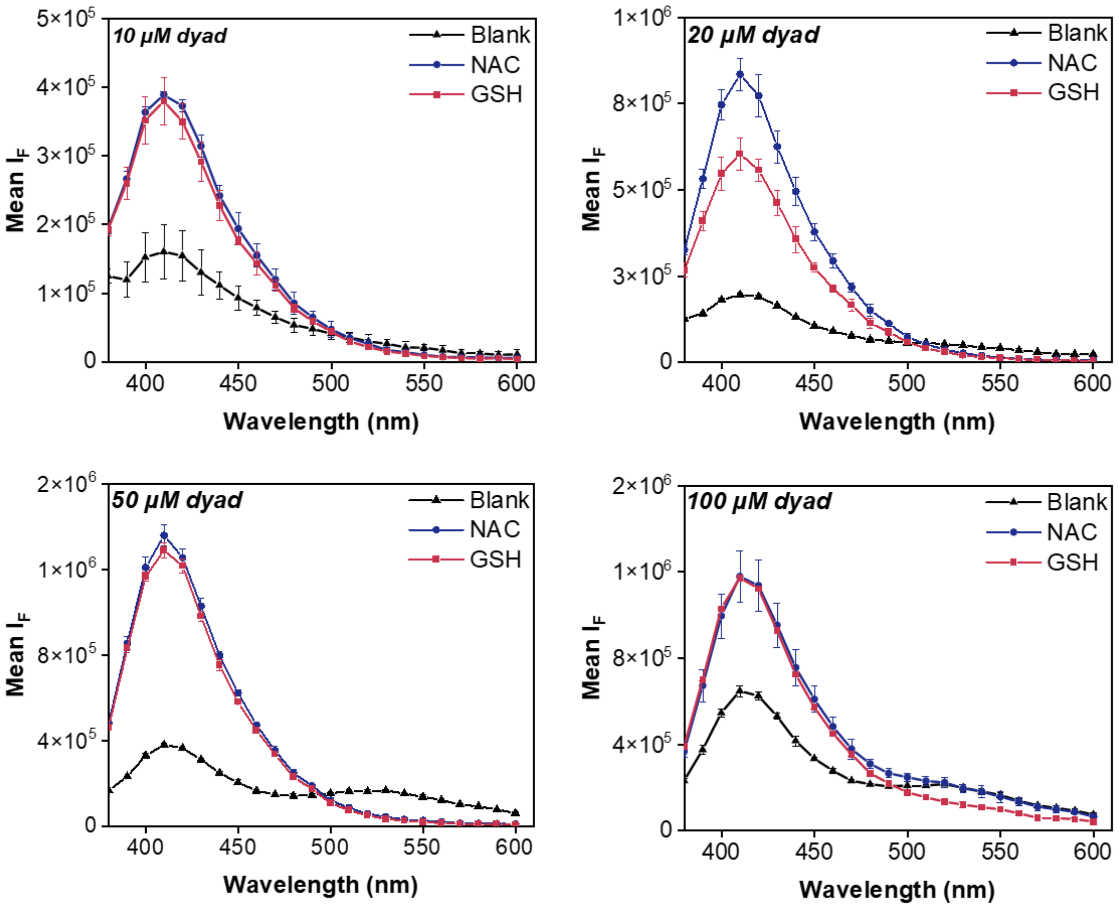

Figure S6. Fluorescence emission spectra of **FRET-Dyad** (10-100 μM) in the absence and presence of 50 eq. NAC or GSH in 50 mM PBS pH 7.4 (10% DMSO) (λ_{ex} = 360 nm, λ_{em} = 380-600 nm incubation time = 1 h). Data are presented as the mean ± SD (*n* = 3).

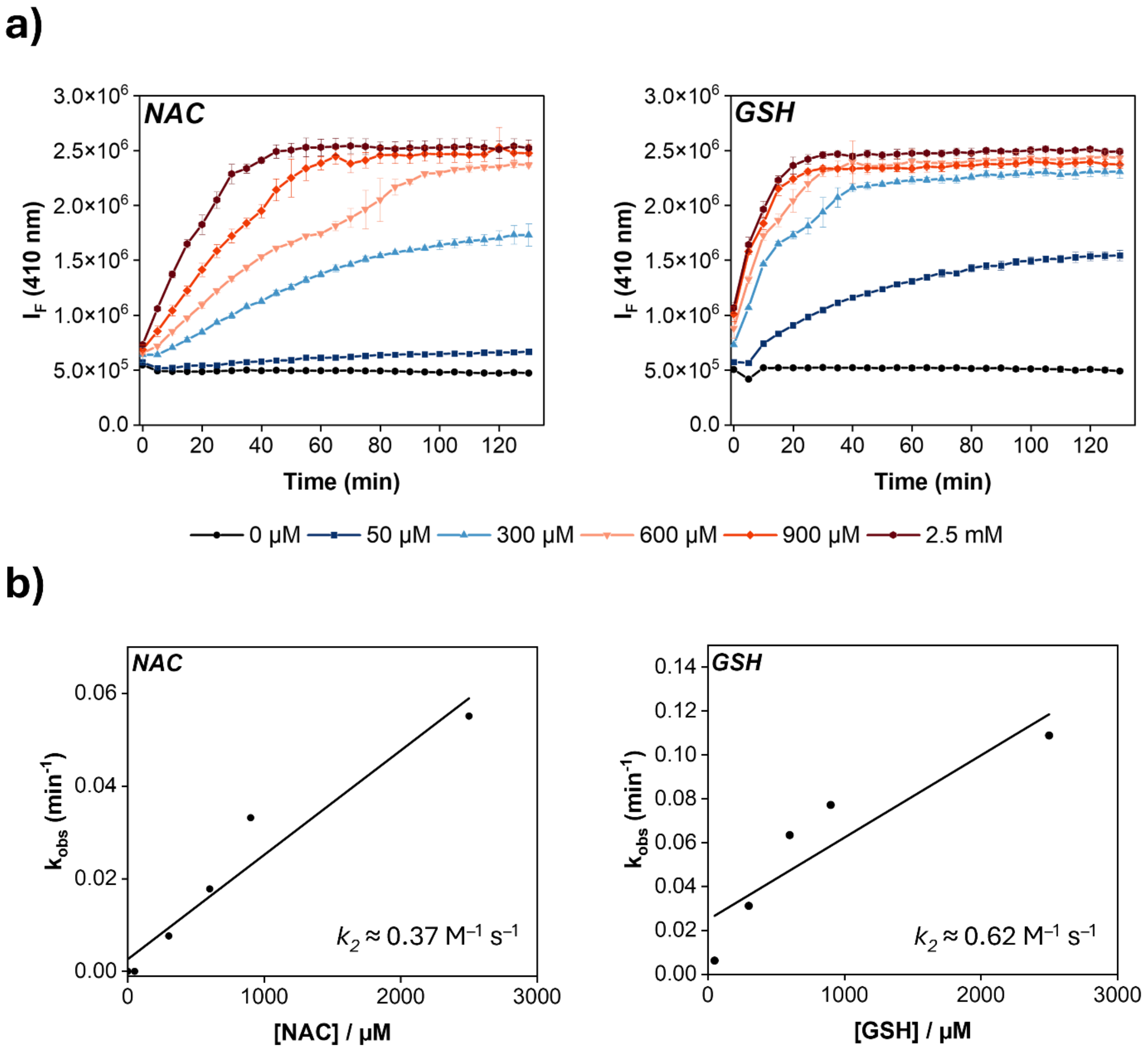

Figure S7. a) Plot of fluorescence intensity change of **FRET-Dyad** as a function of incubation time with different NAC and GSH concentrations (TRIS buffer pH 7.4, rt, 2 h, λ_{exc} = 360 nm, λ_{em} = 410 nm). Data are presented as the mean ± SD (*n* = 4). b) Rates of the fluorescence intensity change of **FRET-Dyad** (50 µM) as a function of NAC or GSH concentration presented in a).

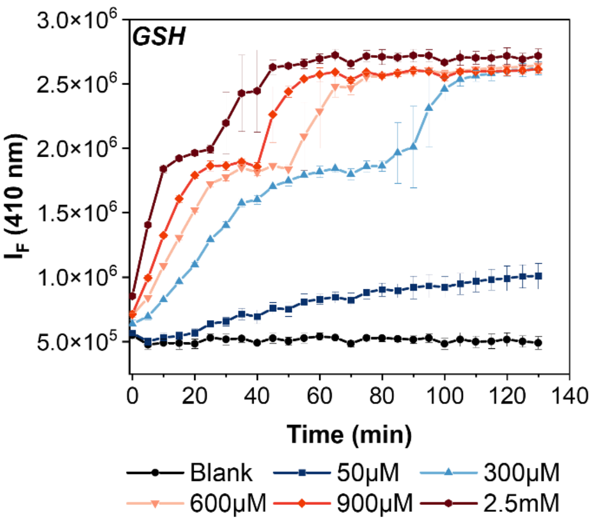

Figure S8. Plot of fluorescence intensity change of **FRET-Dyad** (50 µM) as a function of incubation time with different GSH concentrations (TRIS buffer pH 7.4, rt, 2 h, λ_{exc} = 360 nm, λ_{em} = 410 nm). Data are presented as the mean ± SD (*n* = 4).

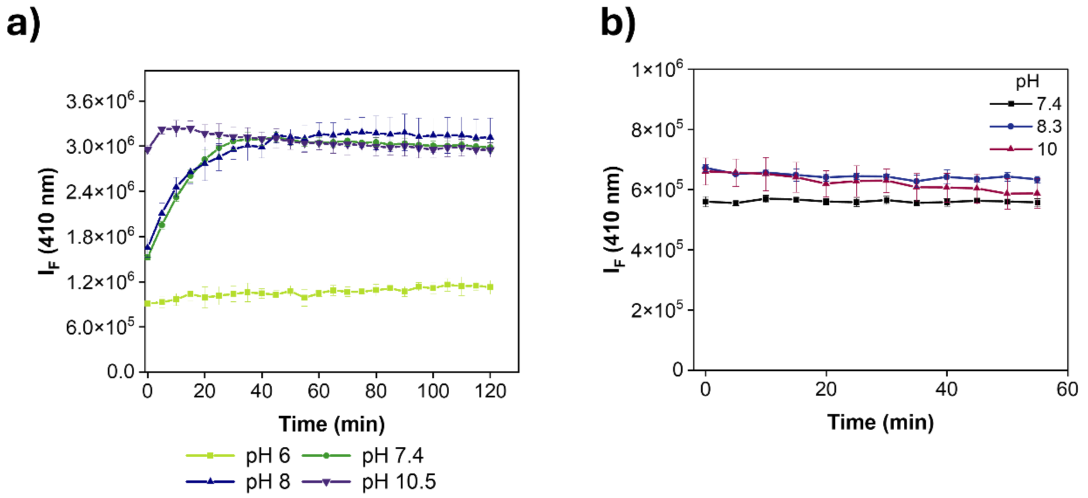

Figure S9. a) Plot of fluorescence intensity change of **FRET-Dyad** (50 µM) as a function of incubation time with 2.5 mM GSH in PBS buffer at different pH values. All buffers contained 10% DMSO as co-solvent. (λ_{exc} = 360 nm, λ_{em} = 410 nm). Data are presented as the mean ± SD (*n* = 4). b) Buffer stability assay of **FRET-Dyad**. Dyad (50 µM) was dissolved in 50 mM TRIS pH 7.4-, or 50-mM PBS pH 8.3, or 50 mM PBS pH 10.5. All buffers contained 10% DMSO as co-solvent and the I_F at 410 nm was monitored over a period of 1 h. Data are presented as the mean ± SD (*n* = 4).

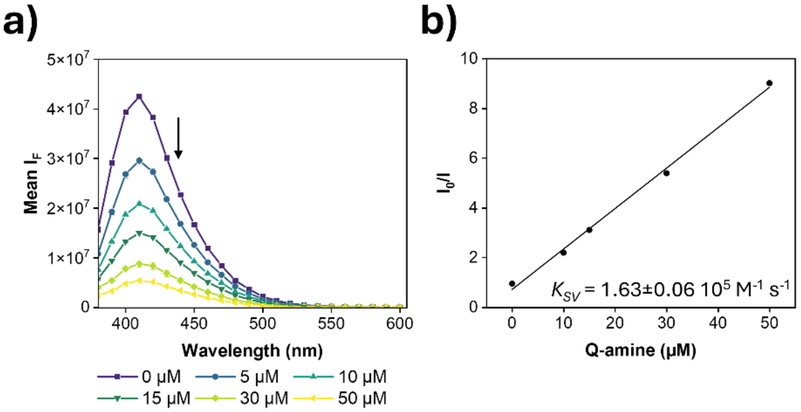

Figure S10. Fluorescence quenching of **Coum** (50 µM) by **Q-amine** (0-50 µM). a) Fluorescence emission spectra of **Coum** (50 µM) at increasing concentrations of **Q-amine** (0-50 µM) in 50 mM TRIS buffer (pH 7.4, 10% DMSO) (λ_{exc} = 360 nm, λ_{em} = 380-600 nm). Data are presented as the mean ± SD (*n* = 3). b) Stern-Volmer plot of the titration experiment, where I_0 corresponds to the fluorescence intensity of **Coum** at 410 nm in the absence of quencher, and I corresponds to that observed in the presence of the quencher.

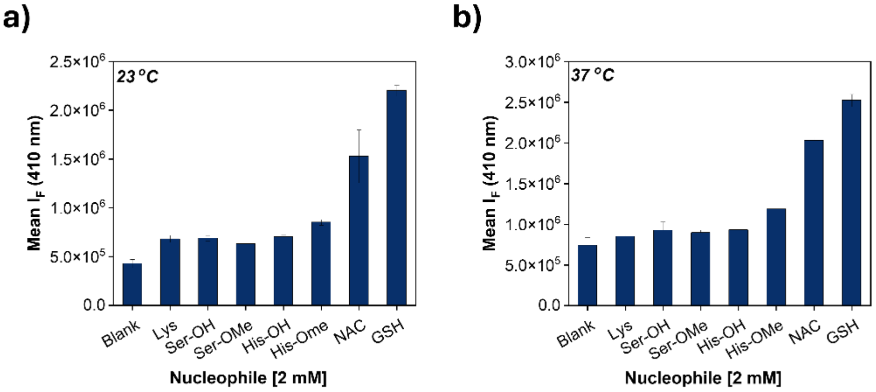

Figure S11. a) Fluorescence responses of **FRET-Dyad** (50 µM) after incubation for 4 h at rt with different nucleophiles (50 eq. λ_{exc} = 360 nm, λ_{em} = 410 nm), 50 mM PBS buffer, pH 8.3 10% DMSO). Data are presented as the mean ± SD (*n* = 4). b) Fluorescence responses of **FRET-Dyad** (50 µM) after incubation for 4 h at 37 °C under the same conditions.

Table S3. Energy of the optimized ground state of **FRET-Dyad** at the SMD(water)/PBE0/6-311+g(d,p) level of theory.

**Energy (Hartrees)**	-2846.277988
**Imaginary frequencies**	0

NMR Spectra

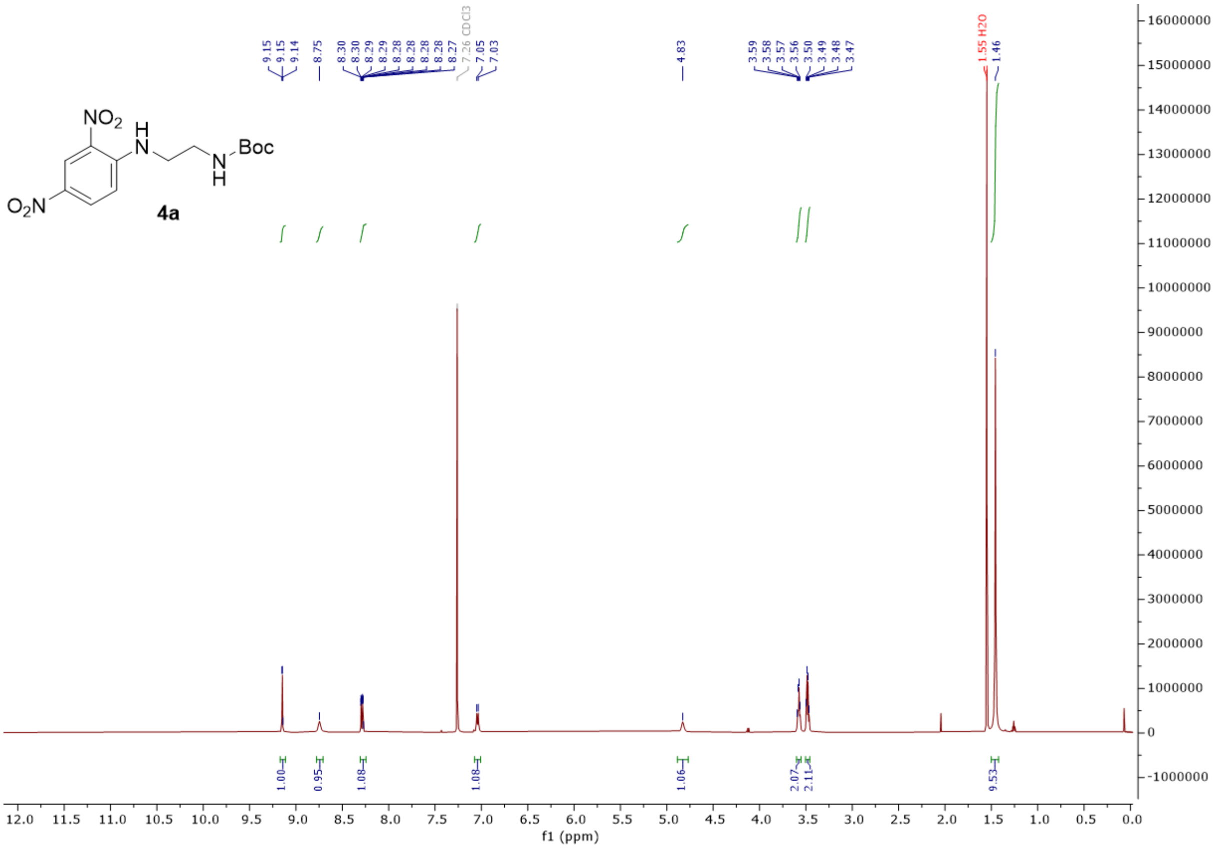

Figure S12. ^{1}H NMR spectrum of compound **4a** (600 MHz, $CDCl_3$).

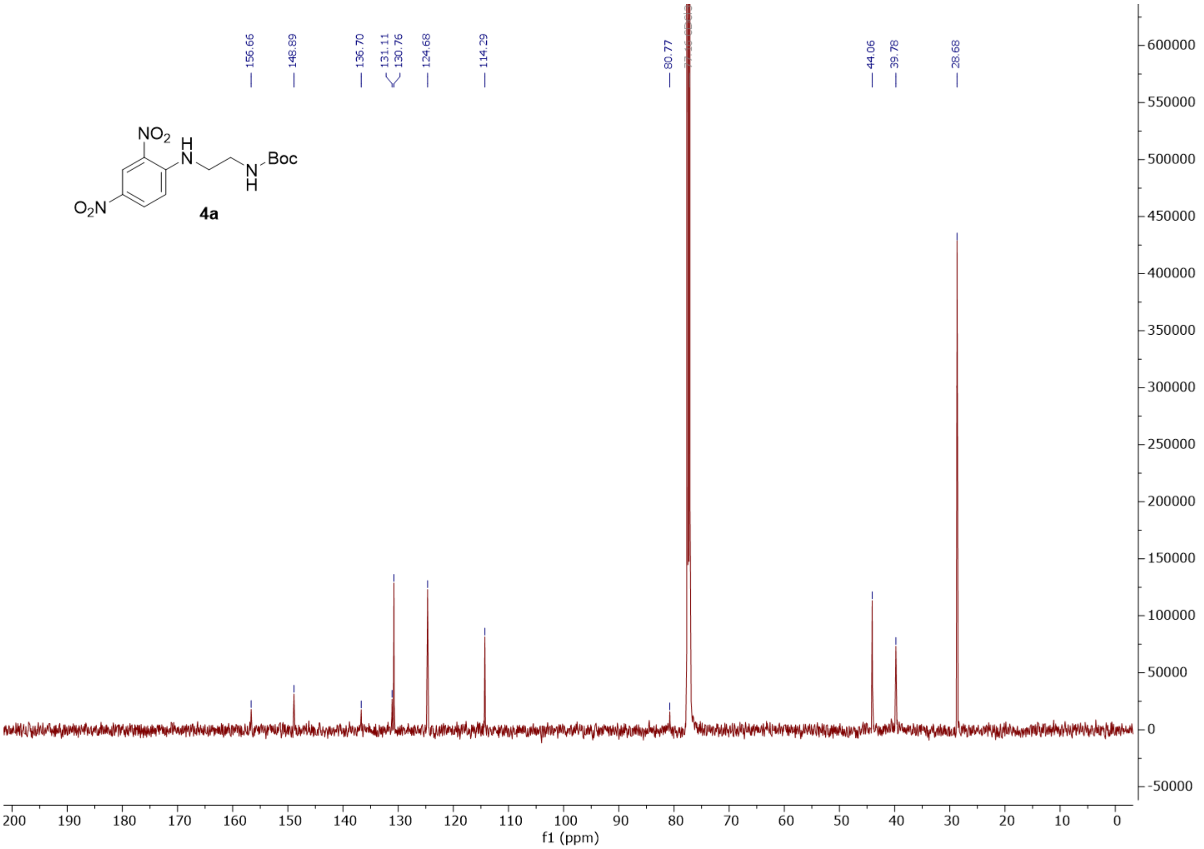

Figure S13. ^{13}C NMR spectrum of compound **4a** (151 MHz, $CDCl_3$).

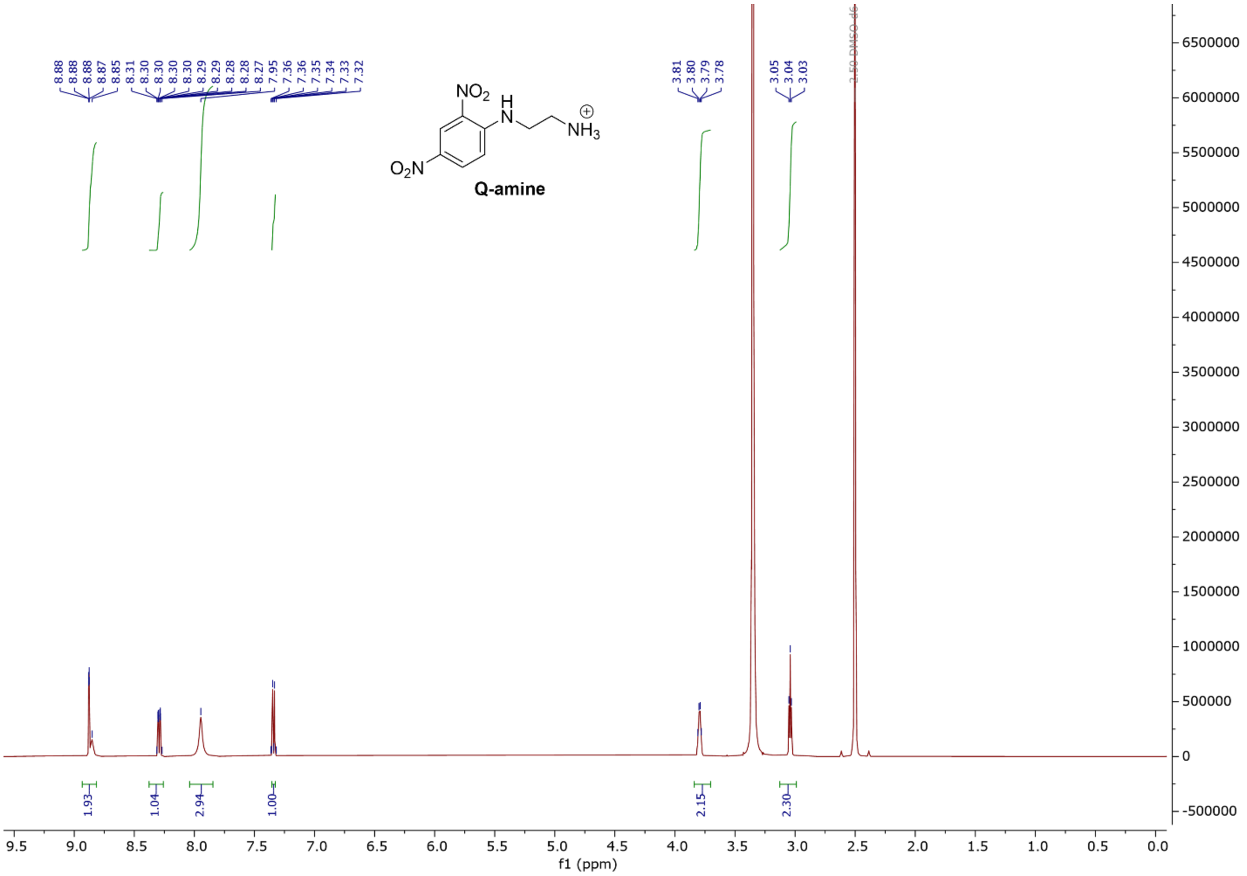

Figure S14. ^{1}H NMR spectrum of compound **Q-amine** (600 MHz, DMSO-d_6).

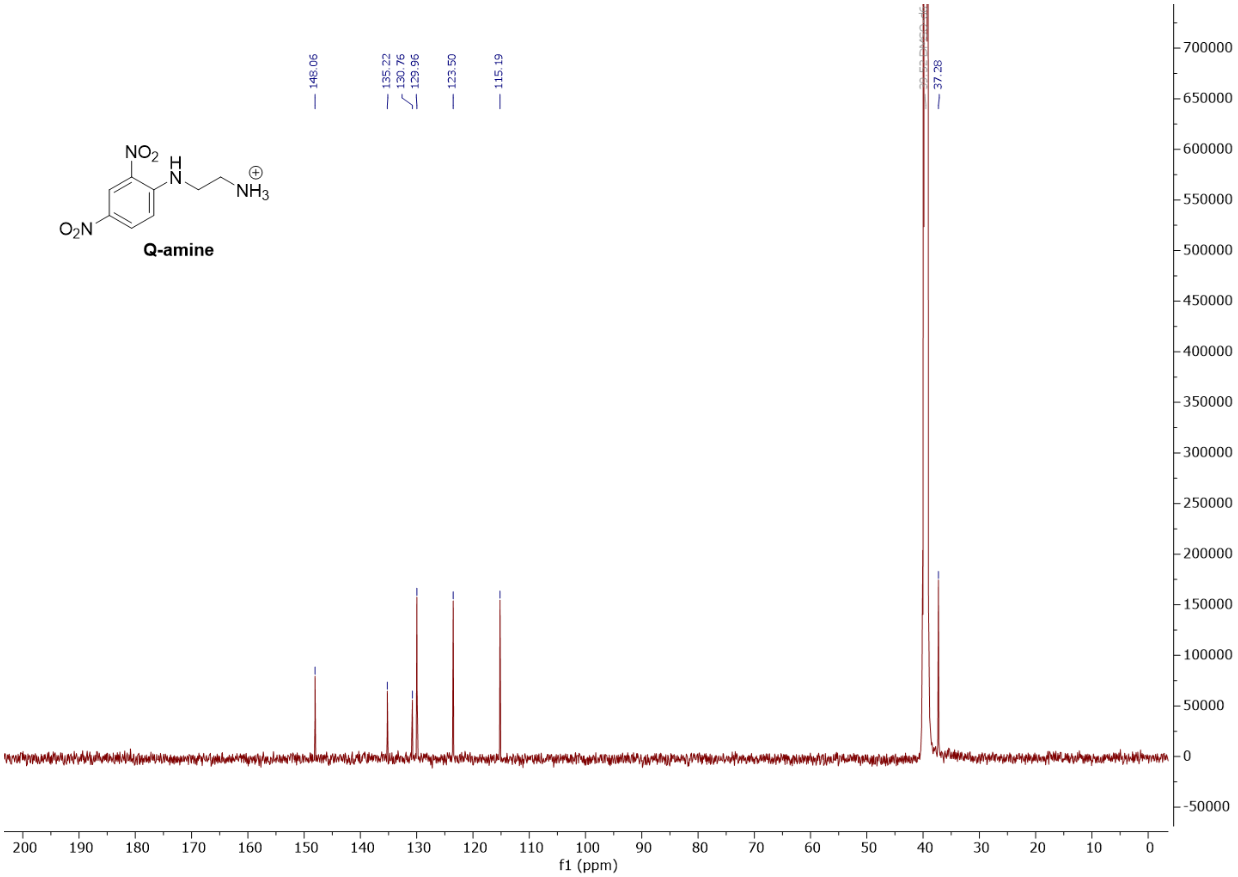

Figure S15. ^{13}C NMR spectrum of compound **Q-amine** (151 MHz, DMSO-d_6).

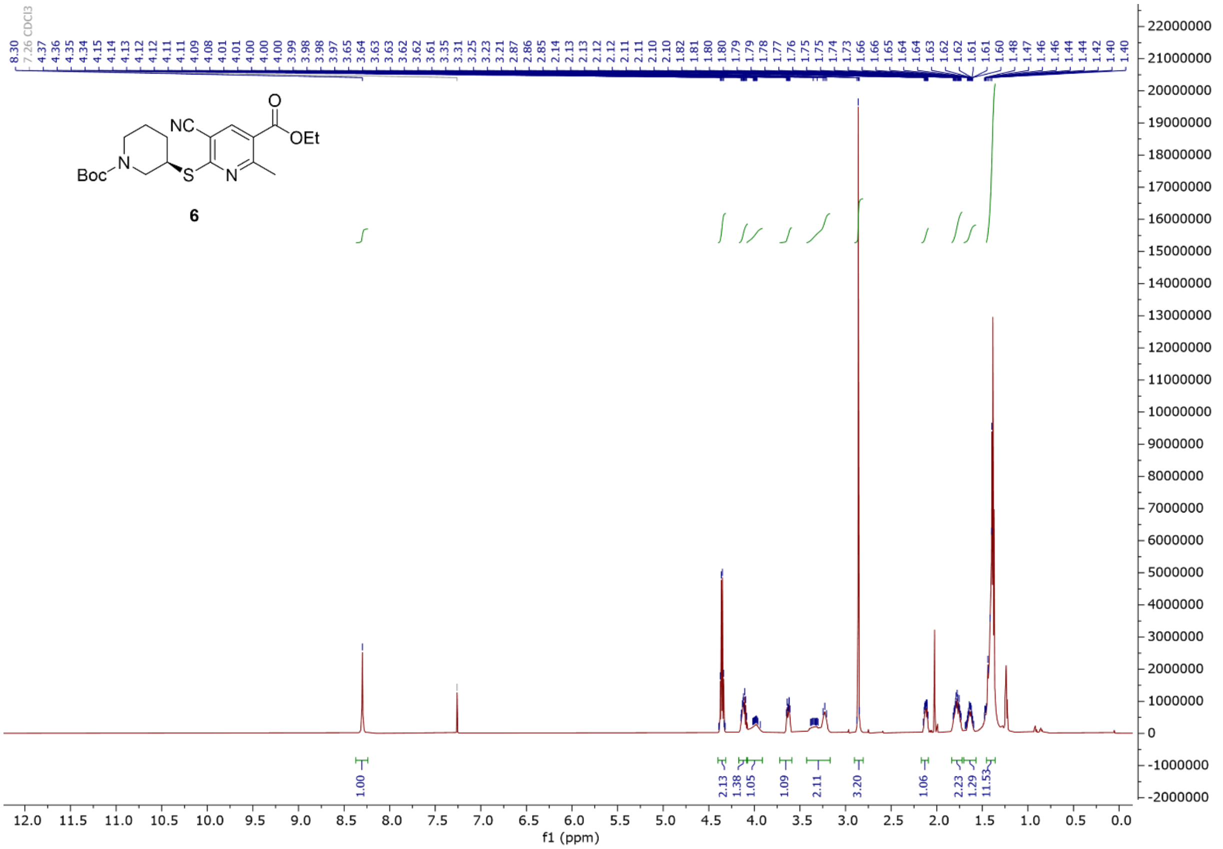

Figure S16. ^1H NMR spectrum of compound **6** (600 MHz, $CDCl_3$).

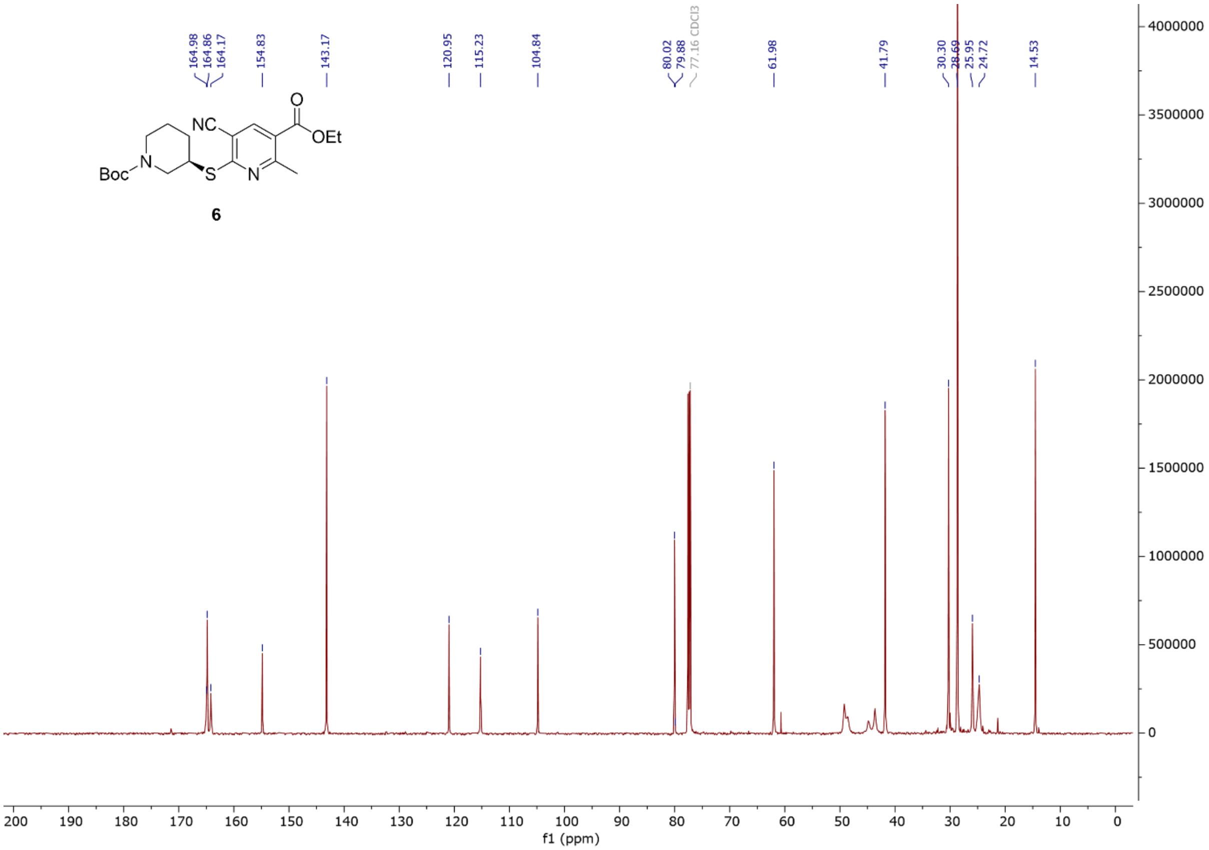

Figure S17. ^{13}C NMR spectrum of compound **6** (151 MHz, $CDCl_3$).

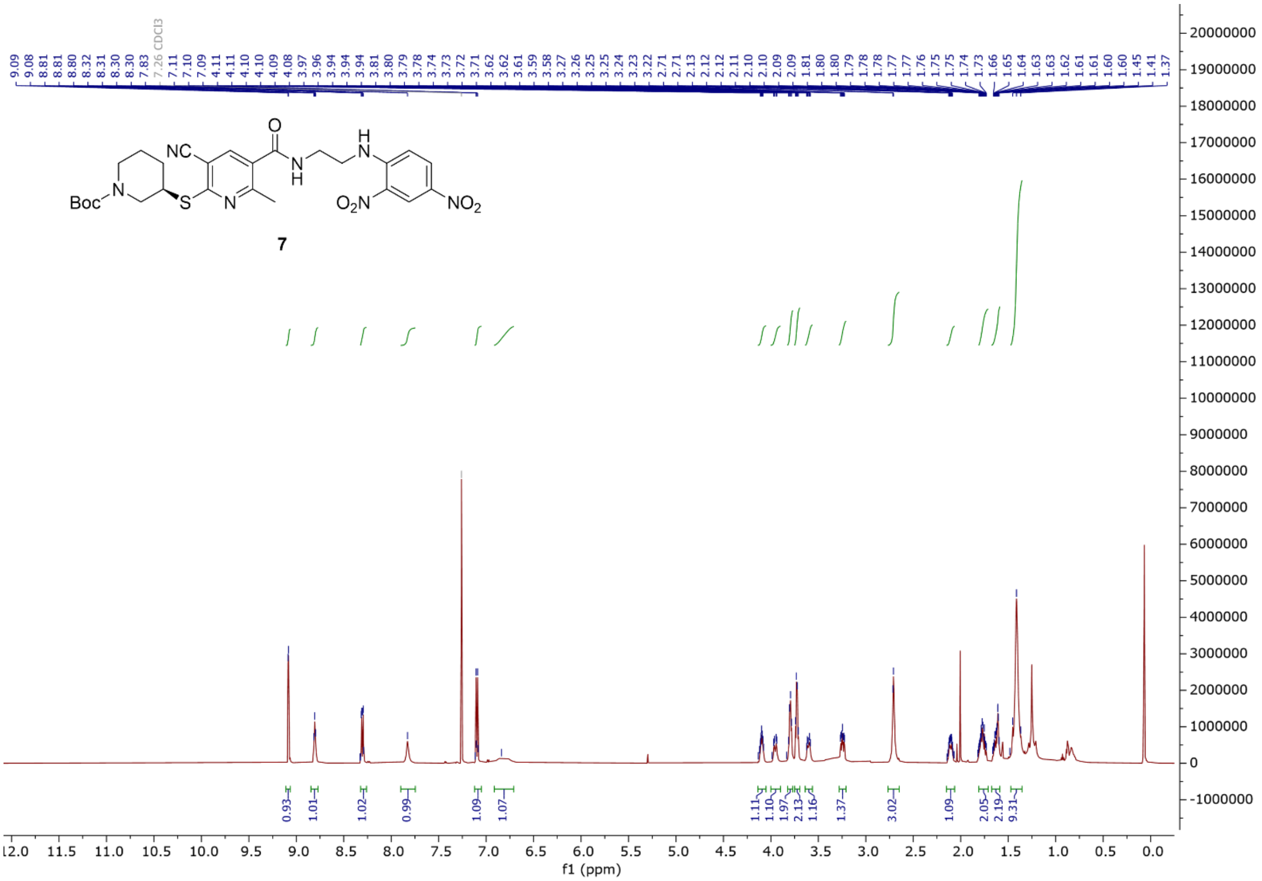

Figure S18. ^{1}H NMR spectrum of compound **7** (600 MHz, $CDCl_3$).

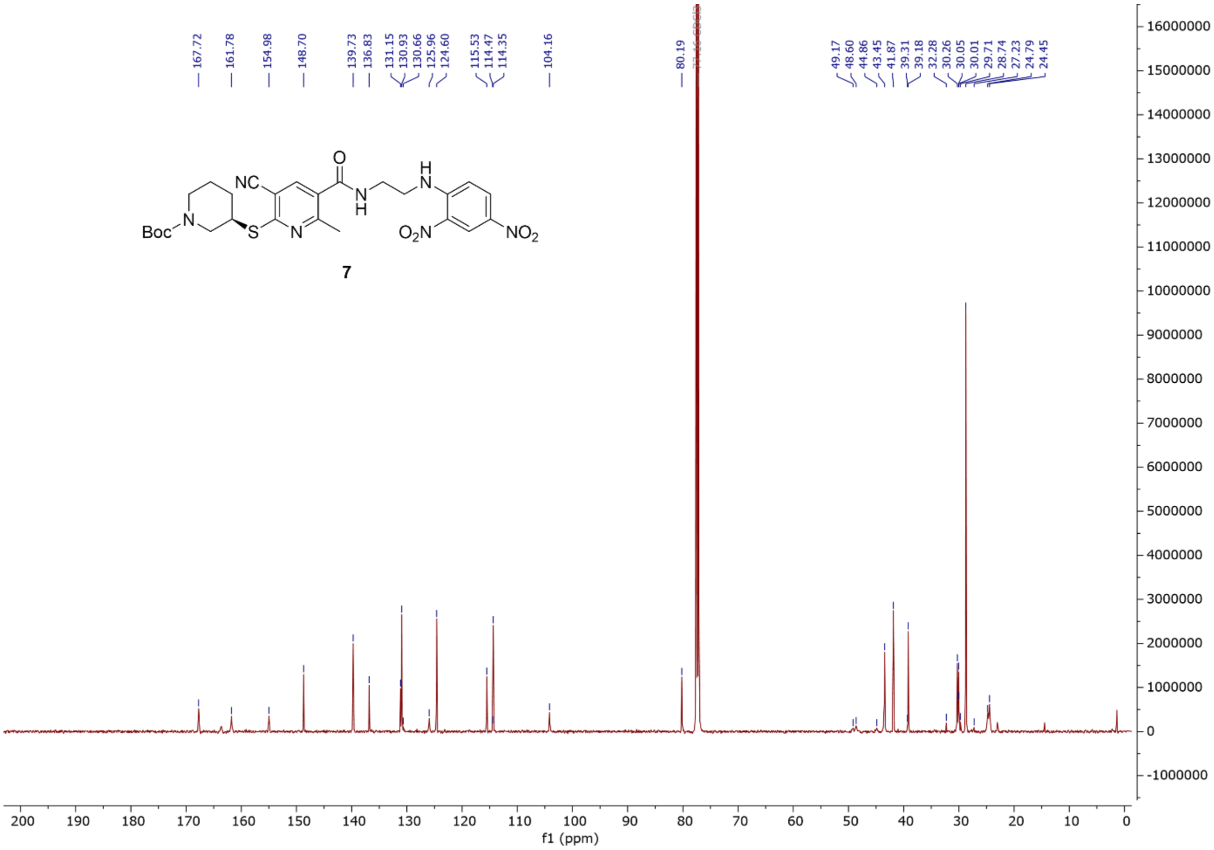

Figure S19. ^{13}C NMR spectrum of compound **7** (151 MHz, $CDCl_3$).

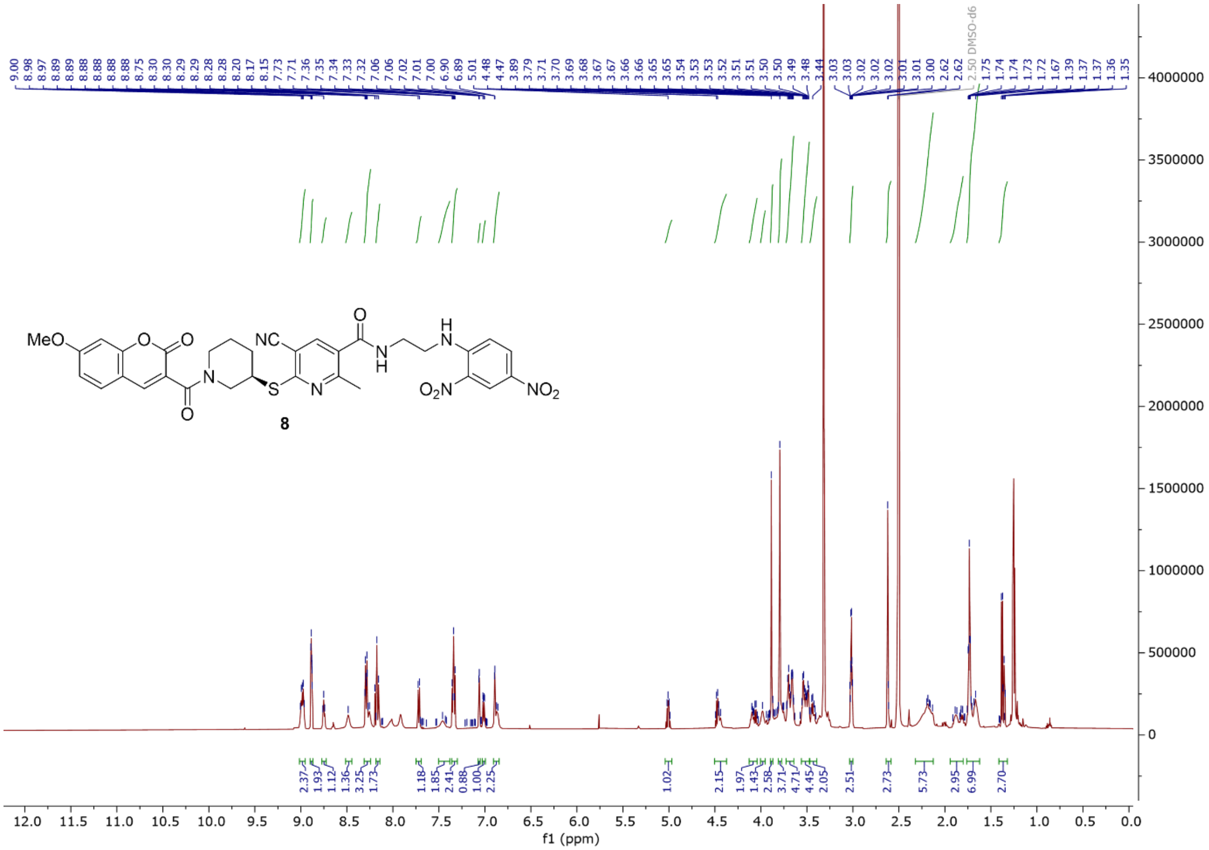

Figure S20. ^{1}H NMR spectrum of compound **8** (600 MHz, DMSO-d_6).

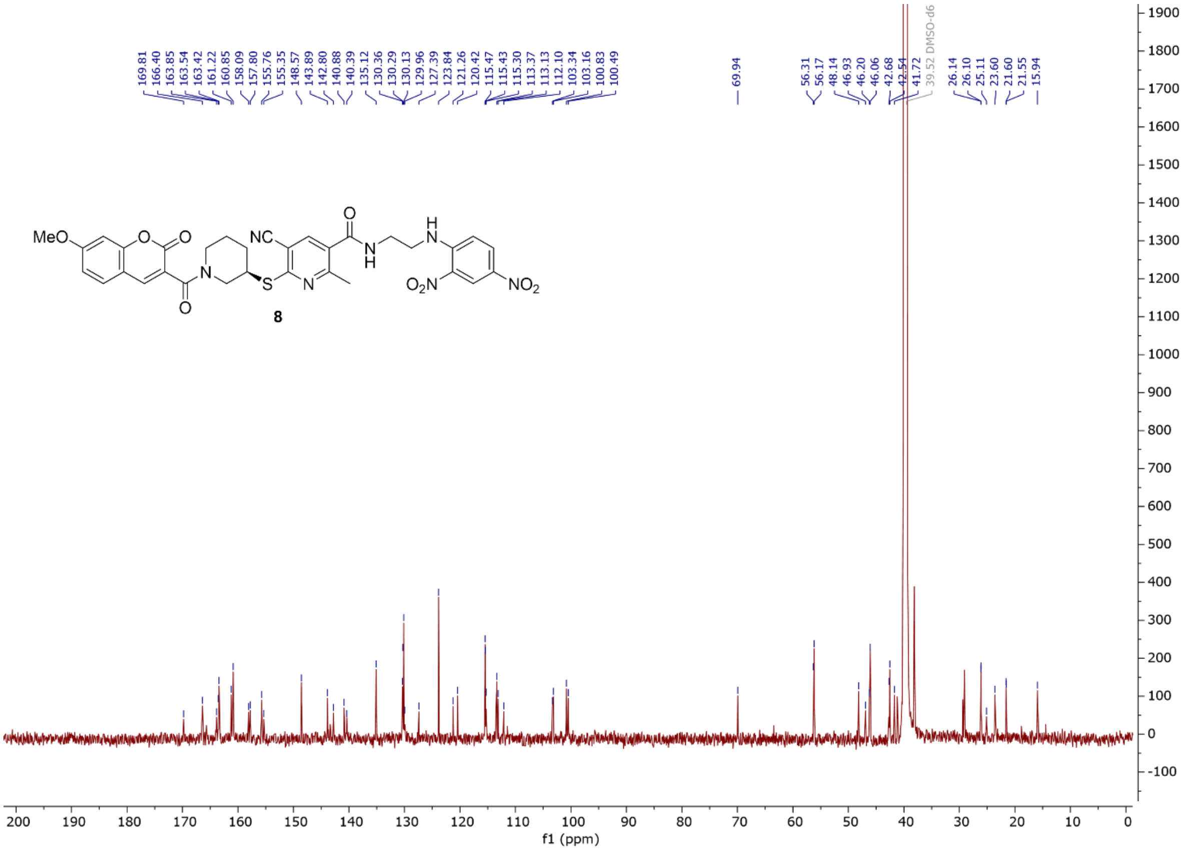

Figure S21. ^{13}C NMR spectrum of compound **8** (201 MHz, DMSO-d_6).

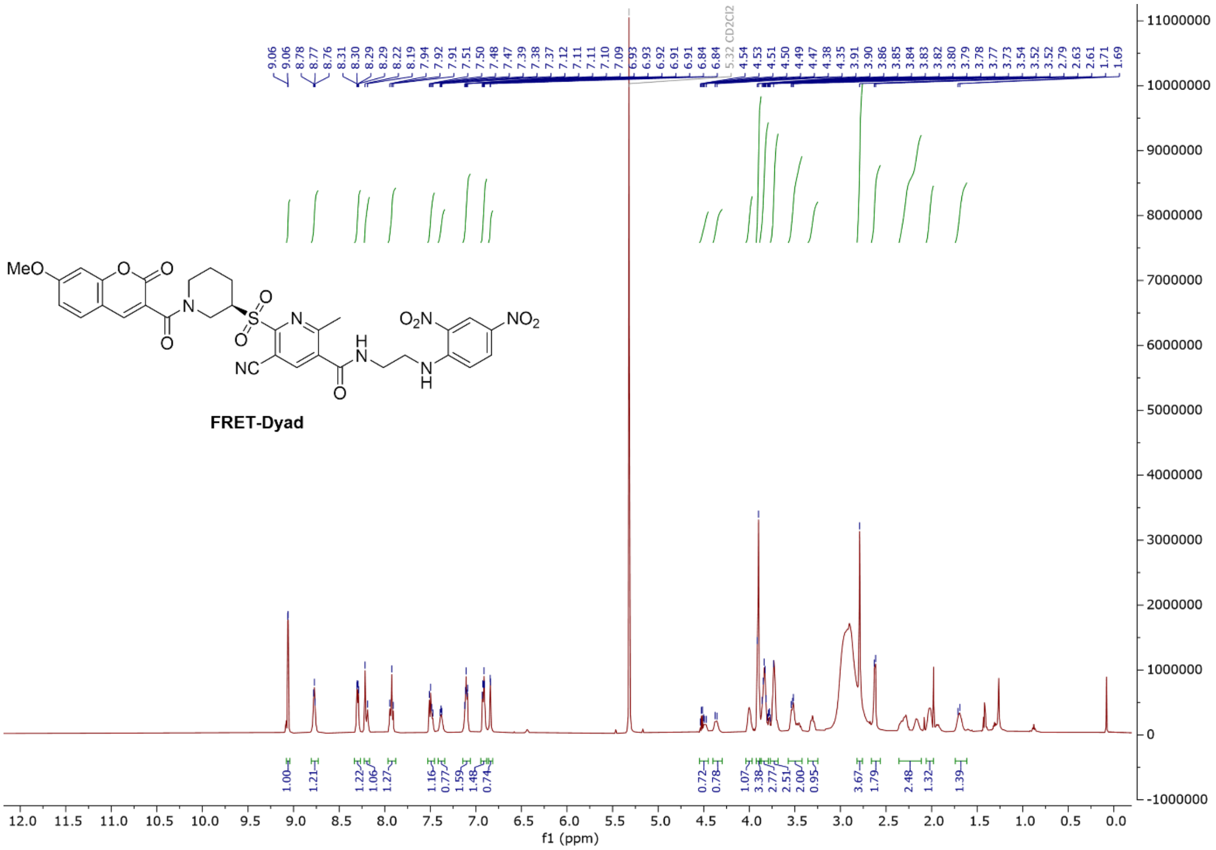

Figure S22. ^{1}H NMR spectrum of **FRET-Dyad** (600 MHz, Dichloromethane-d_2).

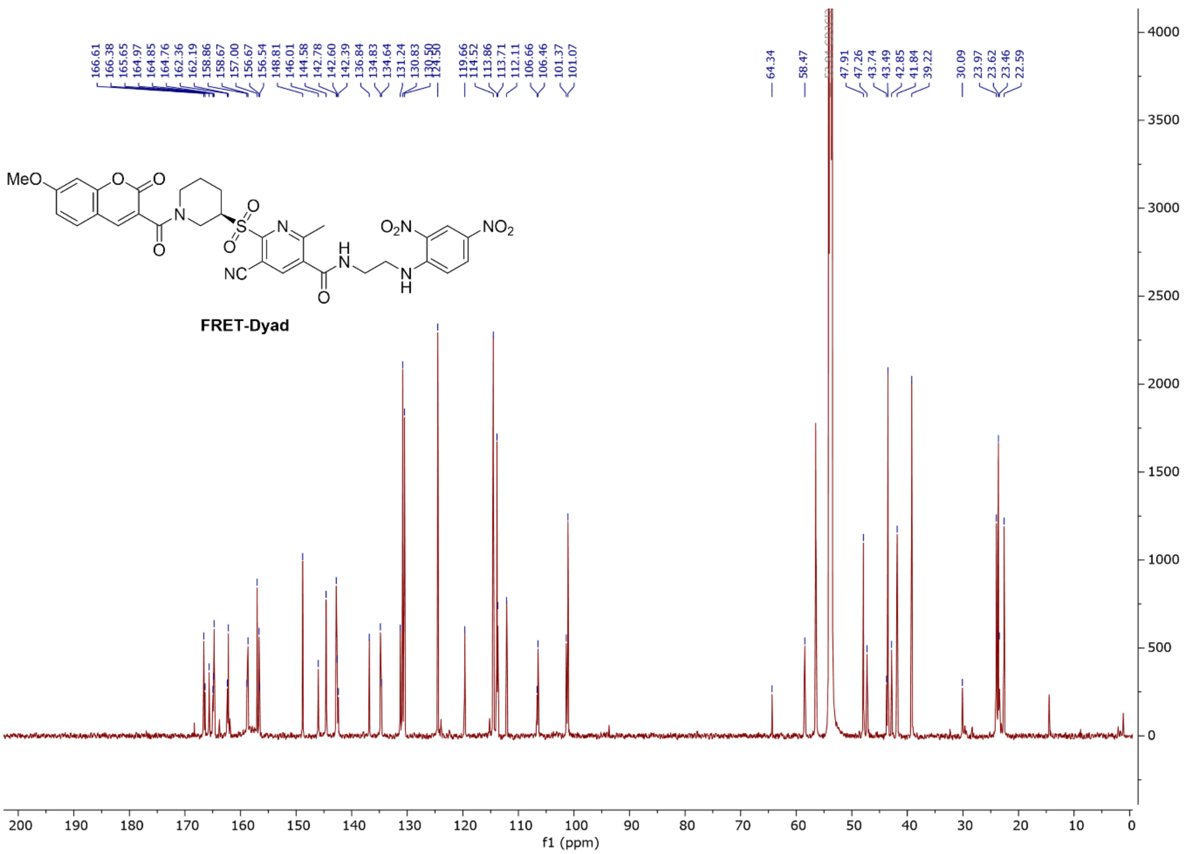

Figure S23. ^{13}C NMR spectrum of **FRET-Dyad** (201 MHz, Dichloromethane-d_2).

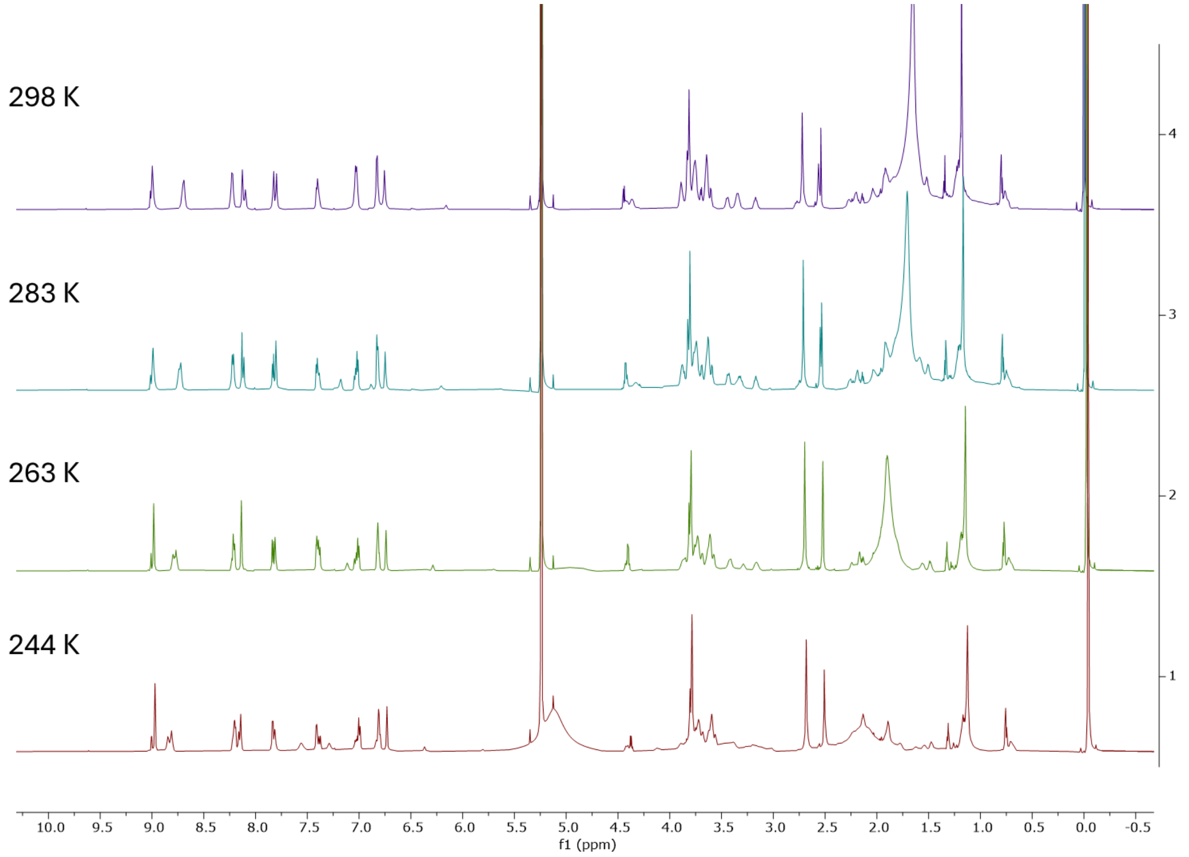

Figure S24. ^{1}H NMR spectra of **FRET-Dyad** (800 MHz, Dichloromethane-d_2) at different temperatures.

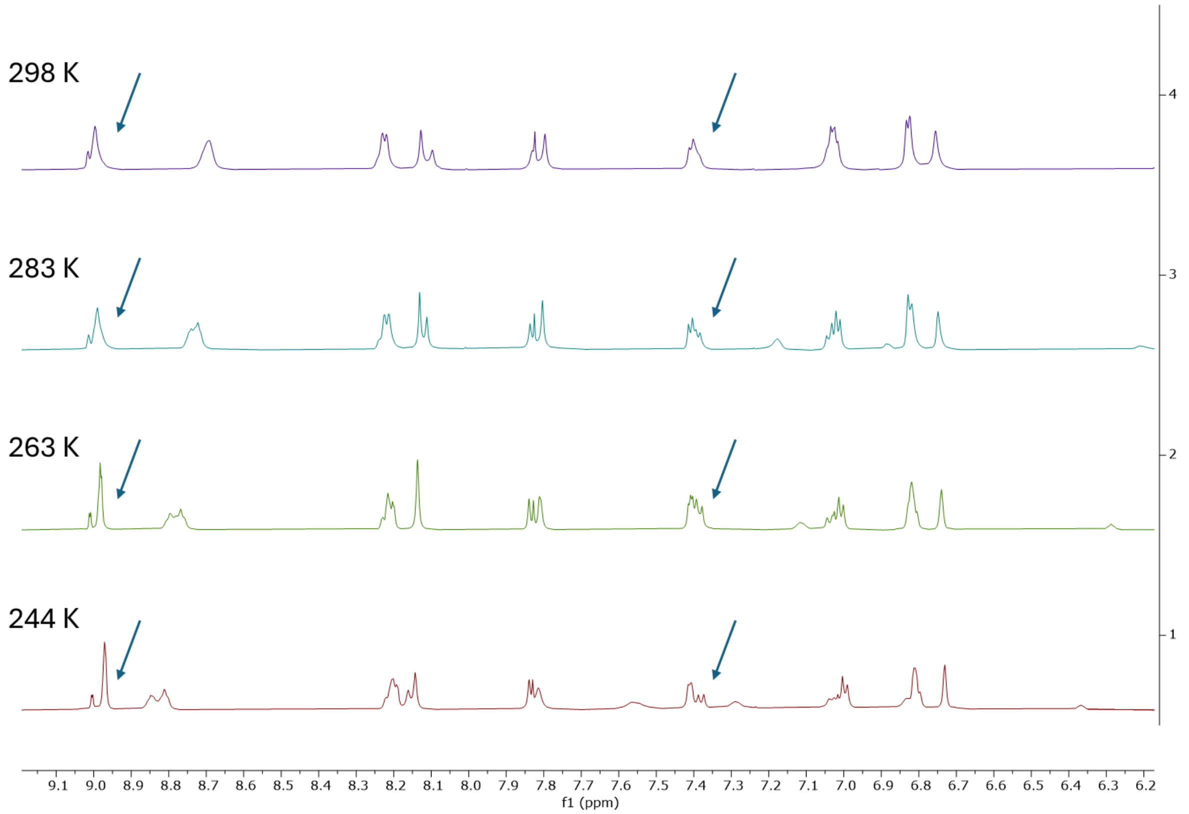

Figure S25. Expanded ^{1}H NMR spectra of **FRET-Dyad** (800 MHz, Dichloromethane-d_2) at different temperatures. The compound exists as rotamers in solution, as proven by NMR. At lower temperatures, slower rotation leads to peak separation, while at higher temperatures an averaged peak is observed. Selected peaks are highlighted with arrows.

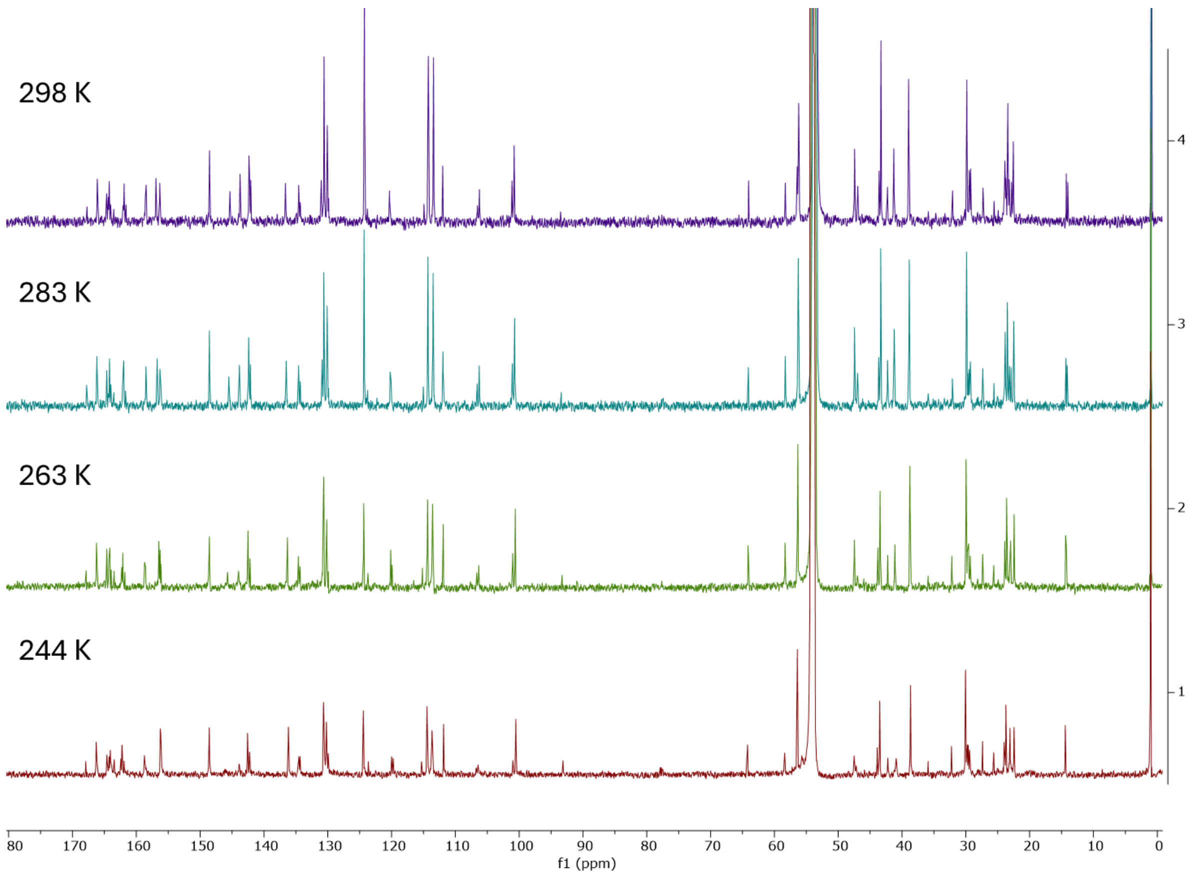

Figure S26. ^{13}C NMR spectra of **FRET-Dyad** (201 MHz, Dichloromethane-d_2) at different temperatures.

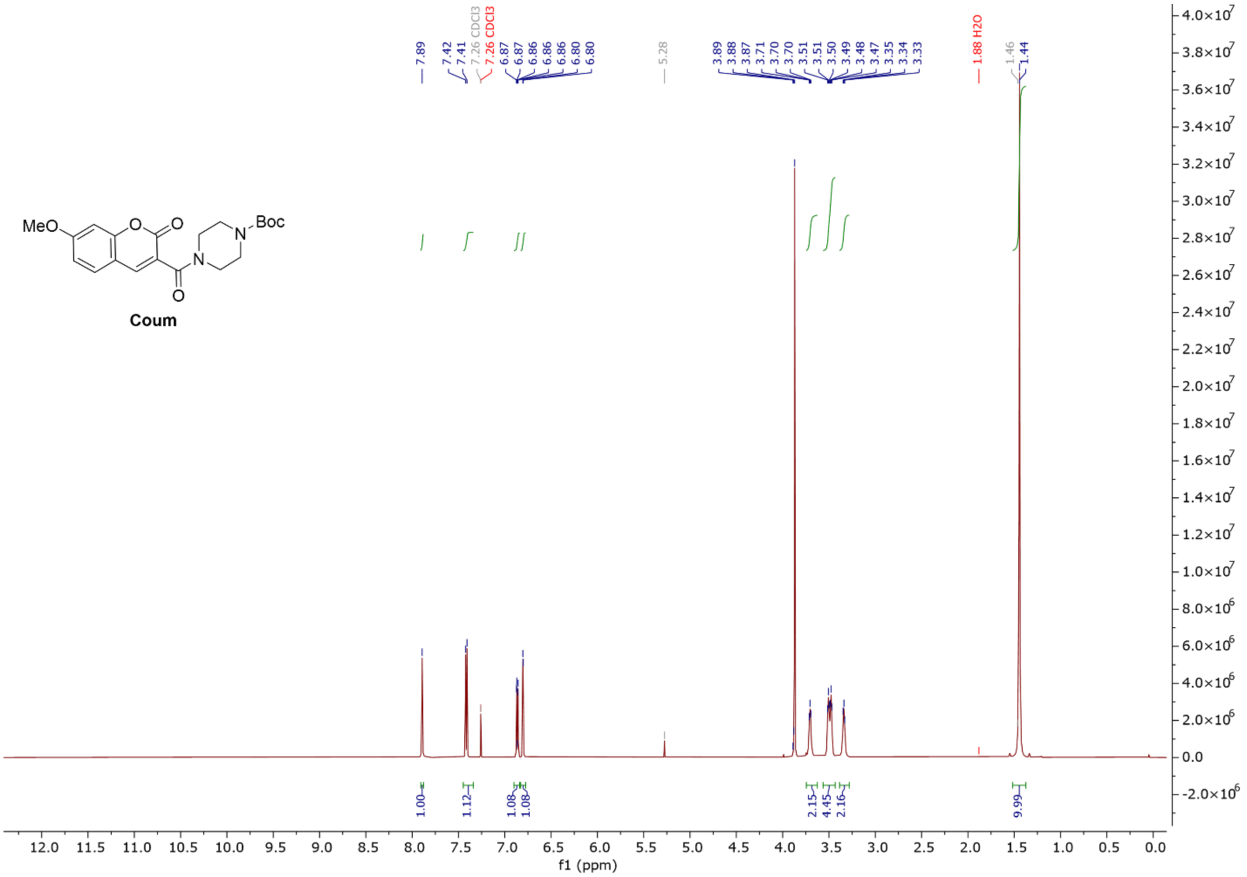

Figure S27. ^{1}H NMR spectrum of **Coum** (600 MHz, $CDCl_3$).

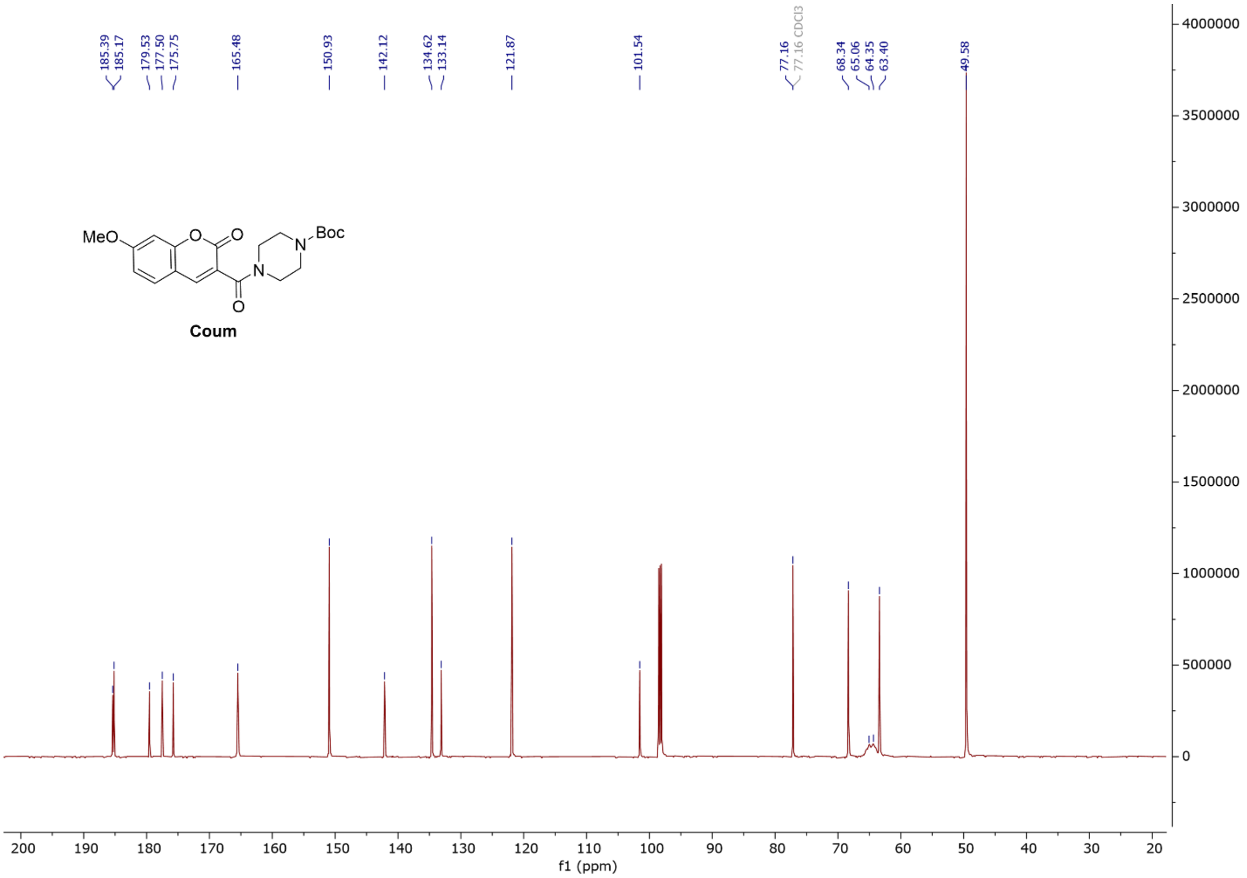

Figure S28. ^{13}C NMR spectrum of **Coum** (151 MHz, $CDCl_3$).

References

(1) Lakowicz, J. R. *Principles of Fluorescence Spectroscopy*, Third Edition, Springer, **2006**.
(2) Markossian S, Grossman A, Baskir H, et al*. Assay Guidance Manual*; Eli Lilly & Company and the National Center for Advancing Translational Sciences, **2004**.
(3) OriginLab - Origin and OriginPro - Data Analysis and Graphing Software, **2023**.
